# Molecular Springs in Dynamic Covalent Polymer Networks

**DOI:** 10.1021/acs.macromol.5c01359

**Published:** 2025-11-11

**Authors:** Ibrahim Oladayo Raji, Mary Eisenhart, Roshan Lama, Govinda Prasad Devkota, C. Scott Hartley, Dominik Konkolewicz

**Affiliations:** Department of Chemistry and Biochemistry, 6403 Miami University, 651 E High St, Oxford, Ohio 45056, United States

## Abstract

Dynamic polymers with remarkable energy dissipation and dampening are highly sought after for advanced applications. Here, spring-like aromatic foldamers were incorporated into the backbones of polymer networks cross-linked with dynamic Diels–Alder (DA) adducts or nondynamic static cross-linkers. Improved mechanical, self-healing, and dampening properties were found in networks with shorter chains, more aromatic stacked foldamer units, and dynamic DA adduct cross-links. These enhanced properties were attributed to the synergistic effects of the reversible elastic response to stress of spring-like aromatic foldamers and the DA adducts.

## Graphical abstract

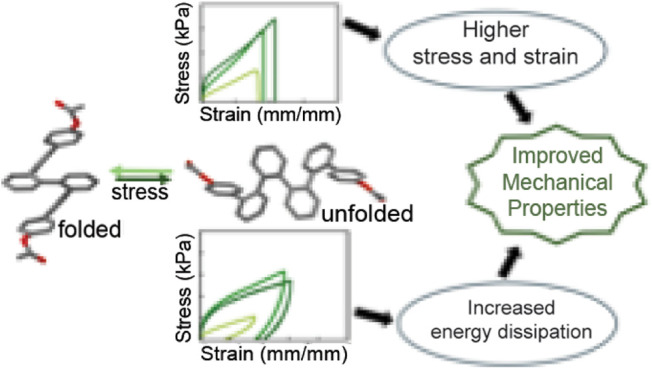

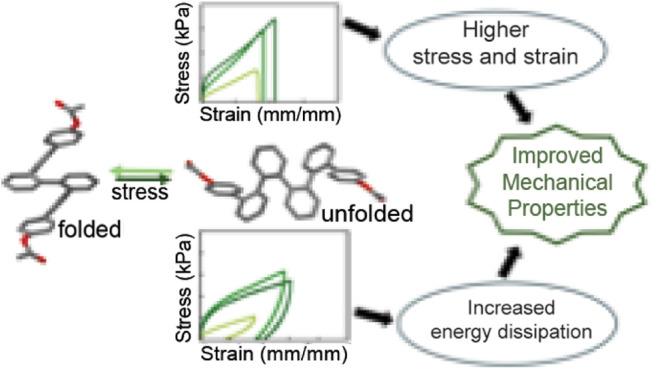

## Introduction

Polymers with excellent dampening, energy dissipation, and self-healing properties are highly desirable due to the increasing environmental noise and vibration pollution from various anthropogenic activities. Developing dynamic and responsive polymers with enhanced energy dissipation and self-healing properties is essential for avoiding material failure and prolonging the lifespans of materials.^1–3^ Nature has often inspired solutions to these challenges in material properties. Many biomaterials have excellent energy dissipation, such as cartilage, which is essential for the function of joints in many animals.^4^ Similarly, the autonomous healing of human skin after minor cuts, with restored sensory and operational functionalities, has inspired self-healing behaviors in polymer networks.^5,6^ Contrarily, many synthetic materials are brittle and can crack or degrade, which reduces their lifetimes. Incorporating dynamic bonds into synthetic materials can enhance not only their self-healing properties but also their toughness and energy dissipation.^7,8^

Over the past decades, significant research has focused on self-healing chemistry through dynamic linkages.^9–11^ Dynamic chemistry, without the use of external healing agents, can be achieved using supramolecular^12,13^ or dynamic covalent linkages.^7,8^ Examples of supramolecular interactions include hydrogen bonds^14^ or host–guest interactions.^15^ Dynamic covalent linkages include thiol-Michael (TM) addition,^16,17^ Diels–Alder (DA) adducts,^18,19^ disulfides,^20,21^ imine formation,^22,23^ and boronic esters.^24,25^ These chemistries have been extensively explored due to their excellent responses to stimuli (i.e., pH, light, heat).^8^ However, there remains an ongoing need for self-healing or energy dissipation in the absence of stimuli for many dynamic systems.

Recently, foldamers have gained attention due to their interesting elastic behavior, which can be harnessed to improve the mechanical properties of polymers, with aromatic foldamers being a particularly useful class of synthetic responsive molecules.^26–31^ Aromatic foldamers are synthetic oligomers with folded molecular conformations that mimic the 3D functions and structures of biomacromolecules, especially proteins.^32,33^ A notable class of aromatic foldamers is *ortho*-phenylenes (*o*Ps), a type of polyphenylene comprising benzene rings that are connected at the ortho positions.^26^ They adopt helical structures through offset aromatic stacking interactions, which are parallel to the helical axis.^26,34^ These folded structures offer a unique synergy between ultimate stability due to covalent bonding and responsiveness due to noncovalent aromatic stacking.^26^

Mechanically responsive materials have several potential applications, ranging from strain sensors to materials with enhanced toughness.^35–38^ Some important strategies have been devised to develop bulk mechanoresponsive polymer materials, including proton catalyst generation,^39–41^ release of small molecules,^42–44^ color changes,^45–47^ isomerization,^48–50^ and incorporation of foldamers, among others.^28–30^ The foldamer-based strategy is promising due to the reversibility of unfolding and folding properties, which enhance energy dissipation.^28–30^ Like proteins, *o*P-based foldamers unwind when stretched at their ends and then promptly rewind upon relaxation.^28^ Therefore, integrating these properties into a polymer network can result in materials with remarkable responses to mechanical forces and excellent energy dissipation.^28^ However, the effects of the incorporation of aromatic foldamers into dynamic polymer networks, coupled with other dynamic covalent bonds, are not well understood.

Herein, we have developed dynamic polymer networks with molecular spring-like aromatic foldamers along the main backbone and thermoresponsive Diels–Alder adduct cross-linkers. This unique strategy presents dynamic polymer networks with increased energy dissipation, dampening, mechanical, and self-healing properties. This innovation is enabled by the ability of the foldamer to unfold and assume unstable elongated configurations upon the application of strain and return to its folded state when relaxed. This is in addition to the thermally driven dynamic exchange of DA linkers, which enables self-healing. This work expands the possibility of harnessing and integrating excellent self-healing properties with folding and unfolding properties into networks to develop dynamic mechanoresponsive materials for emerging technologies in polymer science.

## Results and Discussion

Polymer structure was controlled by a reversible addition–fragmentation chain transfer (RAFT) process. RAFT chain transfer agents (CTAs) containing *o*P units in the center were developed by coupling 2-(propionic acid)­yldodecyl trithiocarbonate (PADTC) with different hydroxyl-terminated aromatic foldamer ortho-phenylene linkers (i.e., *o*P^4^(OH)_2_ and *o*P^6^(OH)_2_) using a 1-ethyl-3-(3-(dimethylamino)­propyl) cardodiimide (EDC) coupling method. *o*P^6^ has six benzene rings and exhibits a double turn of the helix, while *o*P^4^ possesses four benzene rings and has a single turn of the helix. Additionally, a nonfolding CTA based on hydroquinone (HY) was used as a control and synthesized using a similar approach. Subsequently, the difunctional CTAs were used to copolymerize ethyl acrylate (EA) and furfuryl methacrylate (FMA) monomers. EA was utilized as the major backbone-forming monomer due to its elastomeric behavior and relatively low glass transition temperature (*T*_g_), which gives a flexible network that can facilitate dynamic exchange.^18^ FMA was employed as a comonomer to take advantage of the furan motif for possible dynamic DA cross-links in the presence of bismaleimide (BMI). Scheme 1 represents the overall concept of this study and shows the polymer architecture of the dynamic materials.

**Scheme 1:**
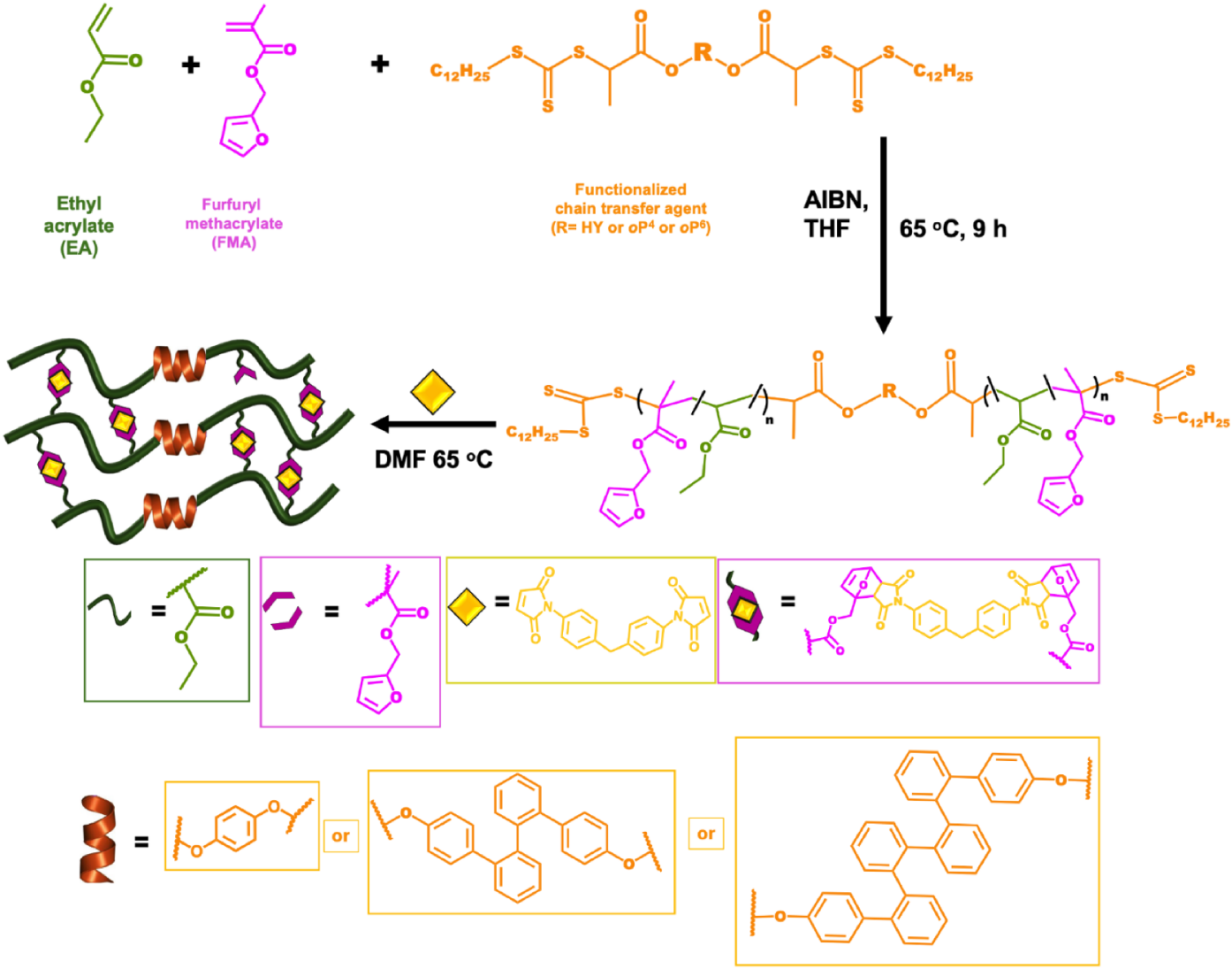
Synthesis of Poly­(EA-FMA) Dynamic DA Cross-Linked Networks Based on Aromatic Foldamer RAFT Agents, *o*P^4^ and *o*P^6^, with Hydroquinone (HY) as the Nonfolded Control

After synthesis of the difunctional CTA with an HY or *o*P core, RAFT copolymerization of EA and FMA was performed at 60 °C for 9 h. Azobis­(isobutyronitrile) (AIBN) was used as the thermal initiator. Well-defined polymers were formed, as shown in the size exclusion chromatography (SEC) plot (Figure S1), with dispersities (Đ) in the range of 1.25–1.45. There is a high molecular weight shoulder in the GPC traces of the poly­(EA-*co*-FMA) polymers. The high molecular weight shoulders seen in the GPC traces in Figure S1 can be attributed to branching formed by polymerization through the diene in pendant FMA units.^51^ This small extent of branching has been seen in prior acrylate/FMA copolymerizations,^19^ but its impact is limited by the preferential reaction between the propagating radical and the acrylic-based monomers. Typical monomer conversions were between 80% and 90% as shown in the ^1^H NMR spectra (Figure S2). After the polymerization was completed, the resulting polymer was purified by precipitation in hexane and dried in a vacuum oven overnight. Postpolymerization cross-linking between the pendant furan groups of the polymer and 1,1′-(methylenedi-4,1-phenylene)­bismaleimide (BMI) was performed. To evaluate the influence of polymer structure on the properties of the synthesized network, a cross-link density of 5% DA adducts (i.e., FMA-BMI) was used for polymers with a degree of polymerization (DP) of 50 or 100. In addition, networks cross-linked with the nondynamic cross-linker ethylene glycol dimethacrylate (EGDMA) were synthesized to separate dynamic DA chemistry effects from aromatic foldamer effects. Hydroquinone-based CTAs were also used as the control for the EGDMA systems, creating materials with neither dynamic DA nor aromatic folding interactions. The polymers synthesized with the FMA comonomer are referred to as HY-FMA, *o*P^4^–FMA, and *o*P^6^–FMA, while networks with the EGDMA cross-linker are referred to as HY-EGDMA and *o*P^4^-EGDMA. Differential scanning calorimetry (DSC) was used to determine the glass transition temperatures (*T*_g_) of the networks. All the synthesized networks have *T*_g_s ranging from −4 to 8 °C (Figure S3), which is below room temperature and consistent with previous works on poly­(EA)-based networks.^18,19^

Tensile tests were used to examine the mechanical properties of the synthesized elastomeric polymer networks. As shown in Figure 1a, polymers with RAFT *o*P linkers incorporated in the backbone have similar extensibility to those with HY backbones, with strain at break (ε_break_) ∼ 1.05 mm/mm to ∼1.1 mm/mm for *o*P^4^ and *o*P^6^, respectively, compared with ε_break_ ∼ 0.9 mm/mm for HY. This could be because the networks have the same DA adduct cross-linkers and the same cross-link density. Further, as the length of the *o*P helix increases, the strength of the polymers with *o*P linkers increases to a peak stress (σ_peak_) of 1200 ± 200 kPa and 1700 ± 300 kPa for *o*P^4^ and *o*P^6^, respectively, compared to 680 ± 40 kPa for materials containing HY at the core of the chain. The extent of aromatic stacking in *o*P^6^ is higher than that in *o*P^4^, while HY has negligible helical aromatic stacking. The increase in strength can be attributed to the unwinding of the folded *o*P units with strain, which could increase the material’s toughness and result in materials that are less brittle. Similarly, Figure 1b indicates that the one-turn *o*P^4^ system has superior mechanical properties in terms of toughness σ_peak_ and ε_break_ compared to the HY-based control. It is important to note that materials cross-linked with nondynamic EGDMA cross-linker are shown in Figure 1b. HY-EGDMA networks have lower ε_break_ (∼ 0.6 mm/mm) and σ_peak_ (at ∼ 360 kPa) compared to *o*P^4^-EGDMA with higher ε_break_ (∼ 0.9 mm/mm) and σ_peak_ (at ∼ 560 kPa). The data in Table 1 and Figure 1 indicate that for both dynamic DA and static EGDMA-based systems, the higher the extent of aromatic stacking, the larger the enhancement in mechanical properties, in terms of both stress and toughness. However, the effect is substantially more pronounced for DA systems compared to the static EGDMA system, perhaps due to the static EGDMA linker reducing effective load transfer to the *o*P-based foldamer

**Figure 1:**
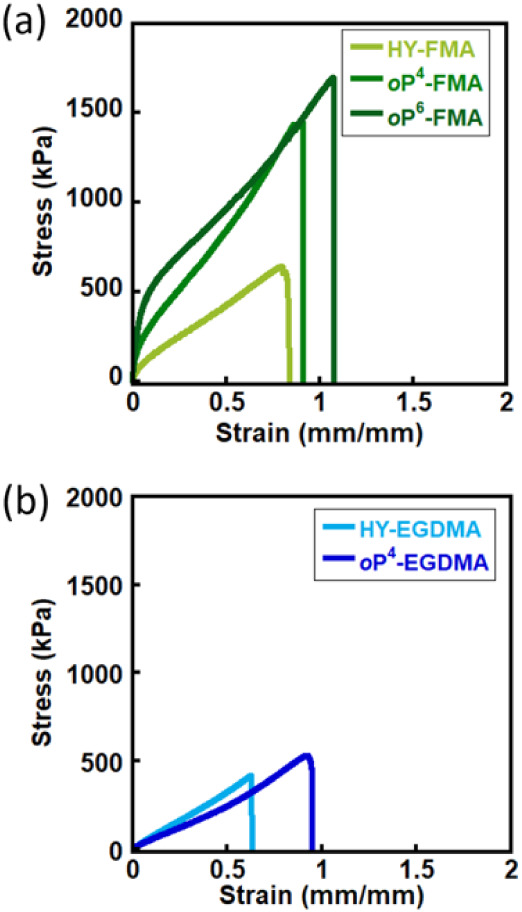
Tensile curve for (a) dynamic cross-linked *o*P^6^–FMA, *o*P^4^–FMA, and HY-FMA and (b) nondynamic cross-linked *o*P^4^-EGDMA and HY-EGDMA synthesized networks.

**Table 1:** Properties of All the Synthesized Poly­(EA) Networks with *o*P^4^, *o*P^6^, and the Control HY

Material	Target DP	σ_peak_ (kPa)	ε_break_ (mm/mm)	Ξ (%) at 0 h	Ξ (%) at 1 h	Ξ (%) at 2 h	*T*_g_	E’_0.01 Hz_ ^a^ (MPa)
HY-EGDMA	50	400 ± 100	0.6 ± 0.1	13 ± 4	13 ± 4	13 ± 5	–4 ± 1	0.19 ± 0.05
oP^4–^EGDMA	50	560 ± 40	0.94 ± 0.1	19 ± 2	20 ± 2	22 ± 4	2 ± 3	0.58 ± 0.08
HY-FMA	50	680 ± 40	0.92 ± 0.07	71.9 ± 0.5	72.8 ± 0.5	71 ± 3	1 ± 2	0.6 ± 0.2
oP^4‑^FMA	50	1200 ± 200	1.1 ± 0.3	84.7 ± 0.4	85.1 ± 0.7	86 ± 1	1 ± 1	0.97 ± 0.05
oP^6‑^FMA	50	1700 ± 300		88.7 ± 0.6	88.2 ± 0.2	89.9 ± 0.3	7 ± 1	1.1 ± 0.3
oP^4‑^FMA	100	600 ± 70	0.8 ± 0.1	26 ± 6	30 ± 7	23.7 ± 0.5	12 ± 4	1.21 ± 0.02

a Storage modulus measured at 35 °C at 0.01 Hz by DMA.

The effect of chain length was also evaluated for networks with the same cross-linker and a cross-link density of 5% (DA adducts) but different chain lengths of DP 50 and DP 100. As shown in Figure S4, a network synthesized with *o*P^4^-based CTA using DA cross-links at a DP of 100 has a relatively small ε_break_ ∼ 0.9 mm/mm and σ_peak_ ∼ 700 kPa compared to a similar network with a DP of 50 with ε_break_∼1.05 and σ_peak_ ∼ 1200 kPa. The higher σ_peak_ observed for DP 50 networks could be due to shorter chains behaving like a denser network, which reduces elasticity and increases stiffness and resistance to deformation, resulting in a higher σ_peak_. DP 100, on the contrary, will have more elastic behavior due to longer chains, hence lower resistance to deformation that leads to lower σ_peak_ in comparison to shorter DP 50 with the same cross-linker and cross-link density. In addition, the higher σ_peak_ observed for DP 50 networks could be due to the same number of cross-links per chain in the shorter chain, which will lead to an effectively higher cross-link density despite having the same cross-link density of 5% DA adducts. However, it is important to note that the spring-like *o*P^4^ linker is in the middle of the chain. Therefore, a longer primary chain also gives a lower effective density of the spring-like *o*P^4^ linker in the material. It is also important to note that the dilution of the *o*P units is particularly significant here because the primary chains are all below the entanglement molecular weight of ∼12,000.^52^

Despite having the same cross-linker and cross-link density, networks with the *o*P-modified CTAs showed enhanced mechanical properties. This can be attributed to the unfolding and folding properties of ortho-phenylene, which can unfold in the presence of strain and refold in the absence of strain. This enables an effective energy dissipation pathway that leads to enhanced toughness. As envisaged, networks with dynamic DA cross-links showed improved tensile properties compared to EGDMA-based networks. This further proves that dynamic DA-based networks can behave mechanically better than nondynamic EGDMA-based networks, due to DA adducts creating less rigid cross-links that support chain mobility and EGDMA creating stiffer cross-links that reduce chain mobility, hence potentially reducing mechanical strength, as reported in the literature.^18,19,53,54^

Self-healing experiments were conducted to examine the dynamic properties of the synthesized networks under thermal stimulus. As shown in Figure 2, the healing characteristics of the aromatic foldamer-based polymers cross-linked with dynamic DA adducts were compared to those cross-linked with EGDMA static cross-linkers. The cut materials were subjected to thermal healing at 90 °C for 24 h and allowed to cool overnight before testing the mechanical properties. The tensile tests showed that materials cross-linked with the static EGDMA cross-linker possess poor healing properties as they were only able to regain ∼10% (Figure 2a and Table S1) of their original mechanical properties, which is attributed to weak van der Waals interactions. In contrast, materials cross-linked with DA adducts were able to regain more than 75% of their original mechanical properties (Figure 2b and Table S1). The self-healing character in the latter can be ascribed to the presence of dynamic DA bonding, which enables thermoresponsive cross-link exchange that facilitates chain mobility to mend the cracks of the cut samples through network reconnection.^18^ EGDMA lacks this bond reconnection potential, hence low chain mobility, which yields materials with poor healing potential.

**Figure 2:**
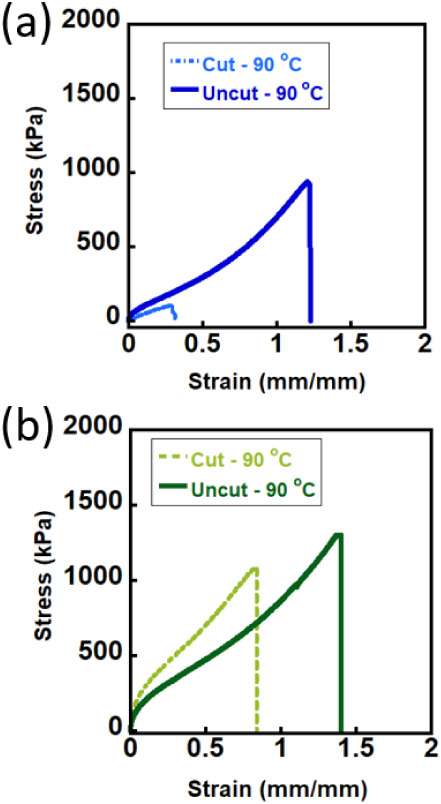
Self-healing tensile curve for (a) nondynamic cross-linked *o*P^4^-EGDMA and (b) dynamic cross-linked *o*P^4^–FMA synthesized polymer networks.

The energy-dissipative potential of the synthesized networks was investigated by cyclic tensile experiments using a universal tensile testing machine at a speed of 3 mm min^–1^ and 30% of the strain at break of each material. Energy dissipation was calculated using the percent hysteresis loop (Ξ), which is a measure of the relative area between the loading and unloading portions of the cyclic stress–strain curve. The hysteresis loop increases with an increasing number of turns in the *o*P system, which shows an indication of increased energy dissipation potential of *o*P-based polymers. The loading–unloading cyclic curve for networks synthesized with aromatic foldamer RAFT CTAs (*o*P^4^ and *o*P^6^), the control (HY), and cross-linked with a dynamic DA adduct is shown in Figure 3a. Networks with *o*P^6^ and *o*P^4^ CTAs showed significantly higher Ξ, as depicted in Table 1 with hysteresis loops of ∼88% and ∼85%, respectively.

**Figure 3:**
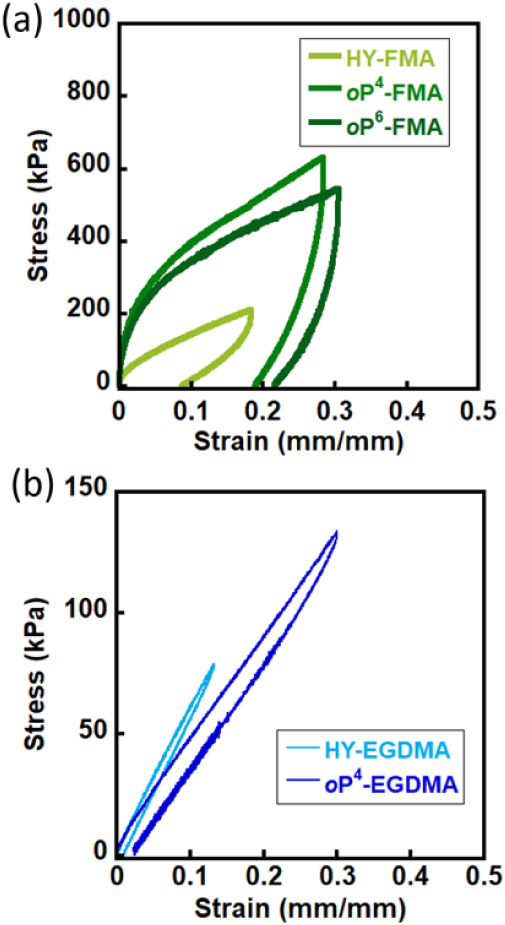
Cyclic tensile curve for (a) dynamic cross-linked *o*P^6^–FMA, *o*P^4^–FMA, and HY-FMA and (b) nondynamic cross-linked *o*P^4^-EGDMA and HY-EGDMA synthesized networks.

Higher Ξ values are envisaged for networks with *o*P CTAs because of their unique unfolding and folding capabilities under strain, which induce spring-like behavior into the networks. The *o*P^6^-based network has slightly higher Ξ than the *o*P^4^-based network, at ∼89% and ∼84% respectively, which can be attributed to the presence of more aromatic stacking interactions from the double-turn spring in *o*P^6^–CTA networks compared to *o*P^4^–CTA networks with a single-turn spring. These networks showed excellent energy dissipation characteristics, which can be attributed to the synergistic effects of the dynamic DA adduct and the *o*P CTAs incorporated into the materials. Polymers with hydroquinone-based RAFT CTA (control) showed notably less energy dissipation (Ξ of ∼71%) compared to polymers with *o*P-based CTAs because the hydroquinone unit has no aromatic stacking interactions in the oP foldamer; hence it is unable to unfold/fold and has no spring-like behavior. There are several possible reasons for this enhanced energy dissipation in the dynamic DA-*o*P foldamer system compared to the control EGDMA-*oP* system. One possibility is that the DA units act as a mechanophore,^55–57^ leading to selective bond breaking and possibly even exchange under mechanical load. Although furan-maleimide units exchange slowly, on the order of hours days at room temperature,^58^ recent literature has shown they can also act as selective bonds for breaking under force.^56^ Alternatively, due to the aromatic structure in the BMI unit and the resulting Diels–Alder adduct, it is possible that these units can assemble and stack in the network,^59^ creating noncovalently bound units that can dissipate energy under load.

To further investigate the recovery properties of the networks, the materials were allowed to rest at 0% strain for 1 and 2 h, and the cyclic tensile testing was carried out again. The results are shown in Figure S5a and b for 1 and 2 h (of rest at 0% strain), respectively. The *o*P-CTA (*o*P^4^ and *o*P^6^) and hydroquinone-based networks all demonstrated a remarkable recovery of the initial loading curve. This result implies the reversibility of the deformation sustained due to chain relaxation, in addition to the refolding of the *o*P units from the cyclic tensile curve after a resting interval, leading to a restoration of the mechanical properties of the network materials. Four repeated cycles without a rest interval between cycles (Figure S6) revealed that energy dissipation potential is predominantly determined between the first loading and unloading curve, with no significant difference in the loading and unloading curves of the subsequent 3 cycles. This is consistent with cyclic tensile results reported in our previous work.^28^

To further understand the mechanical properties of the synthesized networks, the hysteresis of the networks with static EGDMA cross-linkers was also evaluated, as shown in Figure 3b and Table 1. The polymer with a hydroquinone-based CTA showed a lower Ξ of ∼13%, while the polymer with *o*P^4^–CTA showed a somewhat higher Ξ of ∼18%, as envisaged due to the aromatic stacking interactions in *o*P-based networks. Similar to networks cross-linked with dynamic DA adducts, networks with EGDMA also showed excellent recovery of ∼98% to the original loading curve after a resting interval of 1 h (Figure S7a) and 2 h (Figure S7b).

Furthermore, the effect of chain lengths on energy dissipation properties was examined by carrying out cyclic tensile tests on the network synthesized from *o*P^4^–CTA, DA adduct cross-linked, with a DP of 100, as shown in Figure S9. The Ξ is ∼26%, significantly lower compared to the similar network with a DP of 50, which has ∼84% Ξ. This indicates that the energy dissipation effects of the incorporated *o*P^4^ reduce as the chain length increases because of the increased poly­(EA) to *o*P ratio (100:1); hence, minimal effects of *o*P will be observed. Overall, comparing the hysteresis of the networks with static cross-linkers to the networks cross-linked with dynamic DA adducts, it is apparent that the DA-based network showed enhanced energy dissipation properties because of the combined effects of both the DA adduct, which could participate in dynamic exchange, and *o*P, which is able to unfold under strain, hence leading to a higher stress relaxation rate.

Dynamic mechanical analysis (DMA) measurements were performed to examine the damping and viscoelastic properties of the synthesized networks. The DMA experiment was done at a temperature 35 °C, far away from the *T*_g_ of the networks (−4 to 12 °C) to avoid restricted motion induced by a glassy material. DMA was also used to estimate the plateau rubbery modulus, taken as the modulus at 0.01 Hz at 35 °C, of the synthesized networks, as shown in the storage modulus (*E*’) vs frequency plot in Figure 4A,B and Table 1. All of the network materials possess a modulus of 1 MPa at 0.01 Hz and 35 °C, with most systems having a relatively level storage modulus at a low frequency. However, the oP^6‑^FMA system is the least flat in storage modulus over this lowest decade in frequency; however, a storage modulus at lower frequency of ∼1 MPa is consistent with the data in this system. Figure 4C and Table 1 show the damping coefficient factor (tan δ) vs frequency profiles at 35 °C for the *o*P-CTA and HY-CTA in both dynamic and nondynamic cross-linked networks. The dynamic *o*P^6^–CTA-based network with DA cross-links shows the highest tan δ values, hence better energy dissipation compared to dynamic cross-linked *o*P^4^–CTA and HY-CTA-based networks with relatively similar damping potentials. In contrast, only a modest increase in tan δ was seen between *o*P^4^ and HY-based systems in both dynamic Diels–Alder and static EGDMA cross-linked systems. This suggests that the substantially longer spring in *o*P^6^ led to better energy dissipation than the shorter spring in *o*P^4^ or the HY unit.

**Figure 4:**
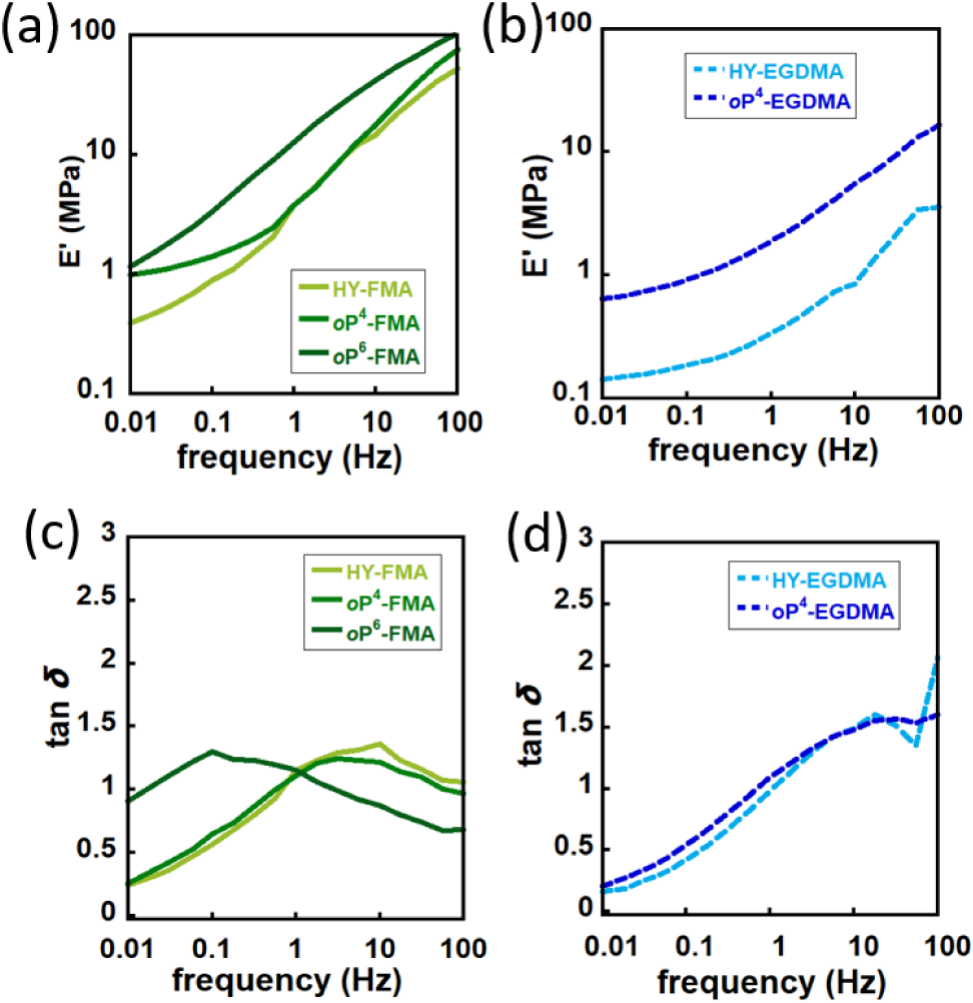
Dynamic mechanical analysis curves of the networks: (a) damping coefficient (tan δ vs frequency) for dynamic cross-linked, (b) nondynamic cross-linked networks, (c) storage modulus E’ vs frequency for dynamic cross-linked, and (d) nondynamic cross-linked networks.

## Conclusion

Polymer networks containing *o*P units in the middle of the chain were developed. Well-defined polymer networks cross-linked by dynamic DA adducts and the static cross-linker EGDMA were synthesized. Networks with *o*P units in the backbone and cross-linked with dynamic DA adducts showed increased mechanical, dynamic, and damping properties compared to networks cross-linked with EGDMA and without *o*P units. The enhanced properties increase as the number of helical turns in the aromatic foldamer increases; hence, *o*P^6^- Diels–Alder adduct-linked materials showed the most significant mechanical and dynamic properties.

## Supplementary Material



## Notes

Experimental methods and procedures, supplemental mechanical testing data, nuclear magnetic resonance and size exclusion chromatography data of polymers, supplemental differential scanning calorimetry data of polymers, supplemental tensile testing data, and melting point data of orthophenylenes (PDF)

## References

[1] Cerdan K., Thys M., Costa Cornellà A., Demir F., Norvez S., Vendamme R., Van Den Brande N., Van Puyvelde P., Brancart J. (2024). Sustainability of Self-Healing Polymers:
A Holistic Perspective towards Circularity in Polymer Networks. Prog. Polym. Sci..

[2] Ekeocha J., Ellingford C., Pan M., Wemyss A. M., Bowen C., Wan C. (2021). Challenges and Opportunities of Self-Healing Polymers and Devices
for Extreme and Hostile Environments. Adv. Mater..

[3] Kim S., Jeon H., Koo J. M., Oh D. X., Park J. (2024). Practical
Applications of Self-Healing Polymers Beyond Mechanical and Electrical
Recovery. Adv. Sci..

[4] Petitjean N., Canadas P., Royer P., Noël D., Le Floc’h S. (2023). Cartilage Biomechanics: From the
Basic Facts to the
Challenges of Tissue Engineering. J. Biomed.
Mater. Res., Part A.

[5] Kashef
Tabrizian S., Terryn S., Vanderborght B. (2025). Toward Autonomous
Self-Healing in Soft Robotics: A Review and Perspective for Future
Research. Adv. Intell. Syst..

[6] Georgopoulou A., Brancart J., Terryn S., Bosman A. W., Norvez S., Van Assche G., Iida F., Vanderborght B., Clemens F. (2022). Soft Self-Healing Resistive-Based
Sensors Inspired
by Sensory Transduction in Biological Systems. Appl. Mater. Today.

[7] Wanasinghe S. V., Dodo O. J., Konkolewicz D. (2022). Dynamic Bonds: Adaptable Timescales
for Responsive Materials. Angew. Chem., Int.
Ed..

[8] Chakma P., Konkolewicz D. (2019). Dynamic Covalent Bonds in Polymeric Materials. Angew. Chem., Int. Ed..

[9] Wool R. P., O’Connor K. M. (1981). A Theory
Crack Healing in Polymers. J. Appl. Phys..

[10] Voyutskii S. S., Vakula V. L. (1963). The Role
of Diffusion Phenomena in Polymer-to-Polymer
Adhesion. J. Appl. Polym. Sci..

[11] Blaiszik B. J., Kramer S. L. B., Olugebefola S. C., Moore J. S., Sottos N. R., White S. R. (2010). Self-Healing Polymers
and Composites. Annu. Rev. Mater. Res..

[12] Li B., Cao P.-F., Saito T., Sokolov A. P. (2023). Intrinsically Self-Healing
Polymers: From Mechanistic Insight to Current Challenges. Chem. Rev..

[13] Roy N., Schädler V., Lehn J.-M. (2024). Supramolecular Polymers:
Inherently
Dynamic Materials. Acc. Chem. Res..

[14] Li Y., Sui J., Cui L.-S., Jiang H.-L. (2023). Hydrogen Bonding Regulated Flexibility
and Disorder in Hydrazone-Linked Covalent Organic Frameworks. J. Am. Chem. Soc..

[15] Bernhard S., Ritter L., Müller M., Guo W., Guzzi E. A., Bovone G., Tibbitt M. W. (2024). Modular and Photoreversible
Polymer–Nanoparticle
Hydrogels via Host–Guest Interactions. Small.

[16] Chakma P., Wanasinghe S. V., Morley C. N., Francesconi S. C., Saito K., Sparks J. L., Konkolewicz D. (2021). Heat- and
Light-Responsive Materials Through Pairing Dynamic Thiol–Michael
and Coumarin Chemistry. Macromol. Rapid Commun..

[17] Grey E. L., McClendon J., Suresh J., Alper S., Janssen W. J., Bryant S. J. (2023). Thiol-Michael
Addition Microparticles: Their Synthesis,
Characterization, and Uptake by Macrophages. ACS Biomater. Sci. Eng..

[18] Dodo O. J., Petit L., Dunn D., Myers C. P., Konkolewicz D. (2022). Thermoresponsive,
Recyclable, Conductive, and Healable Polymer Nanocomposites with Three
Distinct Dynamic Bonds. ACS Appl. Polym. Mater..

[19] Stopler E. B., Dodo O. J., Hull A. C., Weaver K. A., Chakma P., Edelmann R., Ranly L., Zanjani M. B., Ye Z., Konkolewicz D. (2020). Carbon Nanotube
Enhanced Dynamic Polymeric Materials
through Macromolecular Engineering. Mater. Adv..

[20] Raji I. O., Dodo O. J., Saha N. K., Eisenhart M., Miller K. M., Whitfield R., Anastasaki A., Konkolewicz D. (2024). Network Polymer Properties Engineered
Through Polymer
Backbone Dispersity and Structure. Angew. Chem.,
Int. Ed..

[21] Shimizu T., Whitfield R., Jones G. R., Raji I. O., Konkolewicz D., Truong N. P., Anastasaki A. (2023). Controlling Primary Chain Dispersity
in Network Polymers: Elucidating the Effect of Dispersity on Degradation. Chem. Sci..

[22] Fan Q., Tang Y., Sun H., Guo D., Ma J., Guo J. C. S. (2024). Rapid Self-Healing, and Adaptive
Reprogramming Liquid
Crystal Elastomers Enabled by Dynamic Imine Bond. Adv. Mater..

[23] Liu X., Zhang E., Liu J., Qin J., Wu M., Yang C., Liang L. (2023). Self-healing,
reprocessable, degradable,
thermadapt shape memory multifunctional polymers based on dynamic
imine bonds and their application in nondestructively recyclable carbon
fiber composites. Chem. Eng. J..

[24] Ge M., Li Y., Wu L., Liu R., Miao J.-T. (2024). Structural
Design
Strategy of a Biobased Allyl Compound with Dynamic Boronic Ester Bonds
to Form Rigid-Soft Self-Healing Polymer Networks via Thiol–Ene
“Click” Photopolymerization for Integrated Preparation
of a Flexible Device. Macromolecules.

[25] Zhou S., Wang X., Xu Z., Guan T., Mo D., Deng K. R. S.-H. (2024). Highly Conductive and near-Single-Ion Conducting Gel
Polymer Electrolytes Based on Dynamic Boronic Ester Bonds for High-Safety
Lithium Metal Batteries. J. Energy Storage.

[26] Hartley C. S. (2016). Folding
of *Ortho* -Phenylenes. Acc.
Chem. Res..

[27] Kang S.-H., Jin Y.-J., Kwak G. (2020). Thermomechanical Actuator
Based on
a Molecular-Spring Polymer with a Long Alkyl Side Chain. ACS Appl. Polym. Mater..

[28] Miller K. A., Dodo O. J., Devkota G. P., Kirinda V. C., Bradford K. G. E., Sparks J. L., Hartley C. S., Konkolewicz D. (2022). Aromatic Foldamers
as Molecular Springs in Network Polymers. Chem.
Commun..

[29] Shi Z.-M., Huang J., Ma Z., Zhao X., Guan Z., Li Z.-T. (2010). Foldamers as Cross-Links for Tuning the Dynamic Mechanical Property
of Methacrylate Copolymers. Macromolecules.

[30] Talukdar D., Gole B. (2024). Foldamer-Based Mechanoresponsive
Materials: Molecular Nanoarchitectonics
to Advanced Functions. Langmuir.

[31] Qin J., Wang Y., Wang T., Wang N., Xu W., Cheng L., Yu W., Yan X., Gao L., Zheng B., Wu B. (2024). Anion-Coordination
Foldamer-Based
Polymer Network: From Molecular Spring to Elastomer. Angew. Chem., Int. Ed..

[32] Zhang D.-W., Zhao X., Hou J.-L., Li Z.-T. (2012). Aromatic Amide Foldamers:
Structures, Properties, and Functions. Chem.
Rev..

[33] Bindl D., Mandal P. K., Huc I. (2022). Generalizing the Aromatic δ-Amino
Acid Foldamer Helix. Chem. – Eur. J..

[34] Kinney Z. J., Kirinda V. C., Hartley C. S. (2019). Macrocycles
of Higher *Ortho* -Phenylenes: Assembly and Folding. Chem. Sci..

[35] Dang X., Fu Y., Wang X. (2024). A Temperature
and Pressure Dual-Responsive, Stretchable,
Healable, Adhesive, and Biocompatible Carboxymethyl Cellulose-Based
Conductive Hydrogels for Flexible Wearable Strain Sensor. Biosens. Bioelectron..

[36] Bi D., Qu N., Sheng W., Lin T., Huang S., Wang L., Li R. (2024). Tough and Strain-Sensitive Organohydrogels Based on MXene and PEDOT/PSS
and Their Effects on Mechanical Properties and Strain-Sensing Performance. ACS Appl. Mater. Interfaces.

[37] Ni Q.-Y., He X.-F., Zhou J.-L., Yang Y.-Q., Zeng Z.-F., Mao P.-F., Luo Y.-H., Xu J.-M., Jiang B., Wu Q., Wang B., Qin Y.-Q., Gong L.-X., Tang L.-C., Li S.-N. (2024). Mechanical
Tough and Stretchable Quaternized Cellulose Nanofibrils/MXene
Conductive Hydrogel for Flexible Strain Sensor with Multi-Scale Monitoring. J. Mater. Sci. Technol..

[38] Dodo O. J., Raji I. O., Arny I. J., Myers C. P., Petit L., Walpita K., Dunn D., Thrasher C. J., Konkolewicz D. (2023). Dynamic Polymer
Nanocomposites towards Strain Sensors and Customizable Resistors. RSC Appl. Polym..

[39] Klok H.-A., Herrmann A., Göstl R. (2022). Force Ahead:
Emerging Applications
and Opportunities of Polymer Mechanochemistry. ACS Polym. Au.

[40] Liu P., Jimaja S., Immel S., Thomas C., Mayer M., Weder C., Bruns N. (2024). Mechanically Triggered On-Demand
Degradation of Polymers Synthesized by Radical Polymerizations. Nat. Chem..

[41] Wang Z. J., Gong J. P. (2025). Mechanochemistry
for On-Demand Polymer Network Materials. Macromolecules.

[42] Shen H., Cao Y., Lv M., Sheng Q., Zhang Z. (2022). Polymer Mechanochemistry
for the Release of Small Cargoes. Chem. Commun..

[43] Küng R., Göstl R., Schmidt B. M. (2022). Release of Molecular Cargo from Polymer
Systems by Mechanochemistry. Chem. –
Eur. J..

[44] Zeng T., Ordner L. A., Liu P., Robb M. J. (2024). Multimechanophore
Polymers for Mechanically Triggered Small Molecule Release with Ultrahigh
Payload Capacity. J. Am. Chem. Soc..

[45] Basu A., Wong J., Cao B., Boechler N., Boydston A. J., Nelson A. (2021). Mechanoactivation of
Color and Autonomous Shape Change
in 3D-Printed Ionic Polymer Networks. ACS Appl.
Mater. Interfaces.

[46] Qiu W., Scofield J. M. P., Gurr P. A., Qiao G. G. (2022). Mechanochromophore-Linked
Polymeric Materials with Visible Color Changes. Macromol. Rapid Commun..

[47] Liu J., Li W., She Y., Blanchard S., Lin S. (2024). Fatigue-Resistant Mechanoresponsive
Color-Changing Hydrogels for Vision-Based Tactile Robots. Adv. Mater..

[48] Chen X., Shen H., Zhang Z. (2024). Polymer Mechanochemistry
on Reactive
Species Generated from Mechanochemical Reactions^†^. Chin. J. Chem..

[49] Imato K., Ooyama Y. (2023). Polymer Mechanochemistry
of Component Parts for Artificial
Molecular Machines. Macromol. Chem. Phys..

[50] Kean Z. S., Niu Z., Hewage G. B., Rheingold A. L., Craig S. L. (2013). Stress-Responsive
Polymers Containing Cyclobutane Core Mechanophores: Reactivity and
Mechanistic Insights. J. Am. Chem. Soc..

[51] Davidenko N., Zaldívar D., Peniche C., Sastre R., San Román J. (1996). Activity of
the Furfuryl Ring in the Free Radical Polymerization of Acrylic Monomers. J. Polym. Sci., Part A: Polym. Chem..

[52] Andreozzi L., Castelvetro V., Faetti M., Giordano M., Zulli F. (2006). Rheological
and Thermal Properties of Narrow Distribution Poly­(Ethyl Acrylate)­s. Macromolecules.

[53] Vakil J. R., De Alwis Watuthanthrige N., Digby Z. A., Zhang B., Lacy H. A., Sparks J. L., Konkolewicz D. (2020). Controlling
Polymer Architecture to Design Dynamic Network Materials with Multiple
Dynamic Linkers. Mol. Syst. Des. Eng..

[54] Wanasinghe S. V., De Alwis
Watuthanthrige N., Konkolewicz D. (2022). Interpenetrated
Triple Network Polymers: Synergies of Three Different Dynamic Bonds. Polym. Chem..

[55] Cardosa-Gutierrez M., De Bo G., Duwez A.-S., Remacle F. (2023). Bond Breaking of Furan–Maleimide
Adducts *via* a Diradical Sequential Mechanism under
an External Mechanical Force. Chem. Sci..

[56] Stevenson R., De Bo G. (2017). Controlling
Reactivity by Geometry in Retro-Diels–Alder Reactions
under Tension. J. Am. Chem. Soc..

[57] Wang Z., Craig S. L. (2019). Stereochemical Effects on the Mechanochemical Scission
of Furan–Maleimide Diels–Alder Adducts. Chem. Commun..

[58] Diaz M. M., Brancart J., Van Assche G., Van Mele B. (2018). Room-Temperature versus
Heating-Mediated Healing of a Diels-Alder Crosslinked Polymer Network. Polymer.

[59] Safaei A., Terryn S., Vanderborght B., Van Assche G., Brancart J. (2023). Toughening and Stiffening in Thermoreversible
Diels–Alder
Polymer Network Blends. Macromolecules.

