# Supplementary material for: Molecular Springs in Dynamic Covalent Polymer Networks
Source: Macromolecules. 2025 Nov 11;58(22):12346–52. doi: 10.1021/acs.macromol.5c01359 (PMC12659779; doi:10.1021/acs.macromol.5c01359)
Supplement: Supplementary file 1 [file ma5c01359_si_001.pdf]

Supporting Information: Molecular Springs in Dynamic Covalent Polymer Networks

Ibrahim Oladayo Raji[a], Mary Eisenhart[a], Roshan Lama[a], Govinda Prasad Devkota[a], C. Scott Hartley[a] and Dominik Konkolewicz[a].

[a] Department of Chemistry and Biochemistry, Miami University, 651 E High St, Oxford, OH, 45056, USA.

Experimental Procedures

Materials

All materials were obtained from commercial suppliers unless otherwise specified. All materials were used as received unless otherwise specified. Compounds *o*$P^4(OH)_2$ and *o*$P^6(OH)_2$ were synthesized according to the procedures below. All NMR spectroscopy was performed on Bruker Avance 500 MHz or 400 MHz NMR spectrometers. High resolution mass spectrometry was performed at the Indiana University Mass Spectrometry Laboratory using a Thermo Scientific Finnigan LTQ Orbitrap XL Mass Spectrometer using APCI ionization.

1.0 Synthesis of modified CTA and polymer network

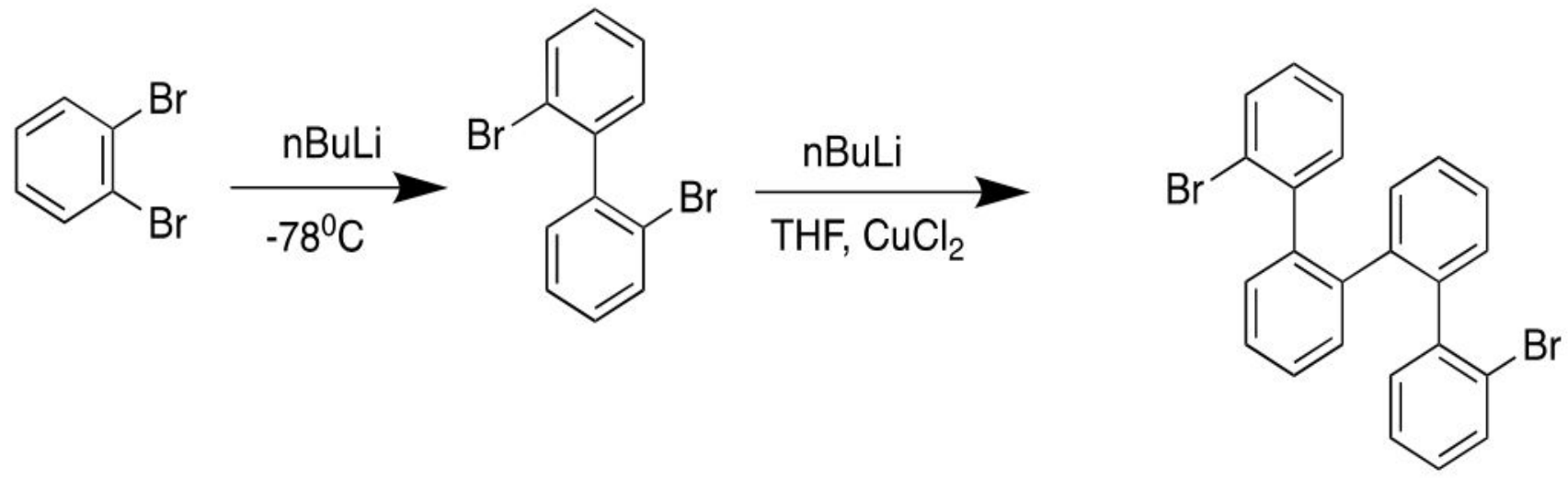

2,2′-Dibromobiphenyl (S1) and Dibromo o-phenylene tetramer (S2) was synthesized according to a literature procedure.[1]

General procedure for Synthesis of dihydroxy *ortho*-phenylenes

1.1 Synthesis of *o*$P^4(OH)_2$

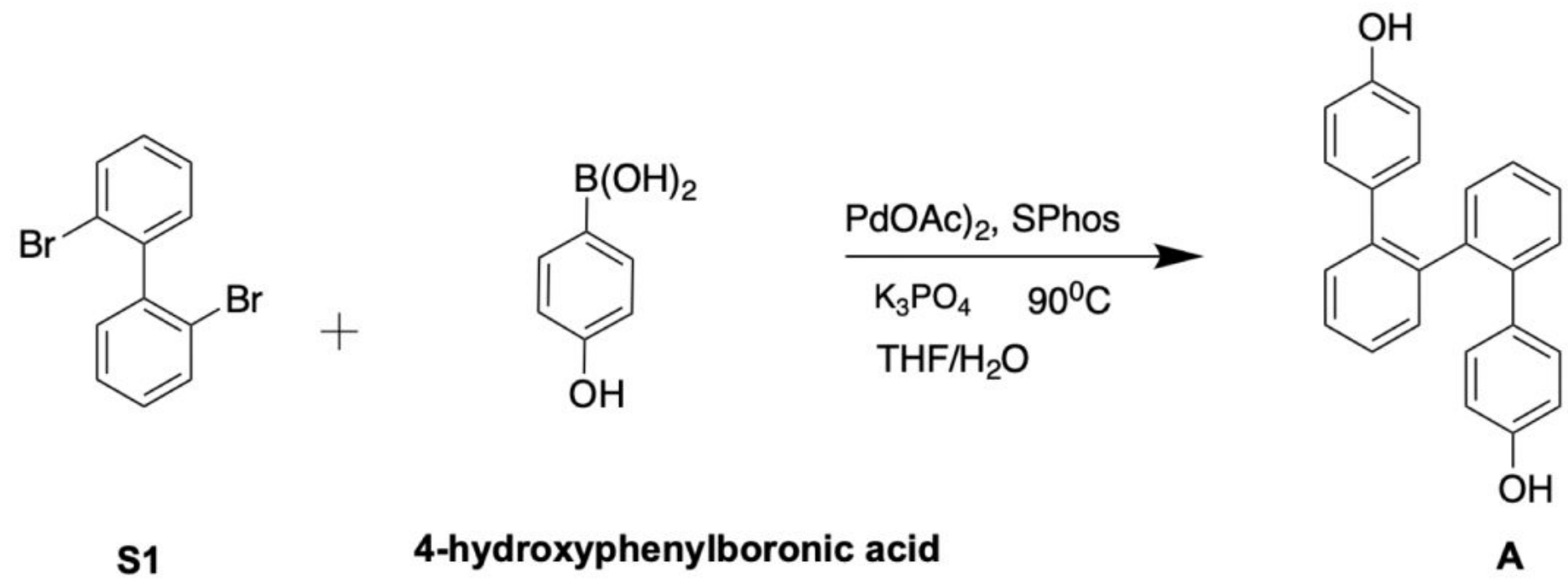

A Schlenk vacuum tube was charged with S1 (1.5 g, 4.80 mmol, 1.0 eq),), 4-hydroxyphenylboronic acid (2.6 g, 19.23 mmol, 4 eq), palladium acetate (258.62 mg, 1.152 mmol, 0.24 eq), SPhos (512.3 mg,1.248 mmol, 0.26 eq), and potassium phosphate (5.0 g, 24 mmol, 5.0 eq) then evacuated and backfilled with argon (3×). Tetrahydrofuran (30 mL) and water (7.5 mL) were then added. The Schlenk tube was degassed by three freeze-pump-thaw cycles, backfilled with argon, then sealed and heated to 90 °C (silicone oil bath) for 24 h. The reaction was then cooled and diluted with ethyl acetate. The organic layer was washed with water (3×) and brine, then dried over $MgSO_4$, filtered, and concentrated under vacuum. The crude products when purified via flash chromatography (1:4 EtOAc:hexanes) to give A as a white solid (1.5g, 92%) mp: 150.90 °C; HRMS (MALDI) calc'd for $C_{24}H_{17}O_2$ 337.1229, found ([M-H$^+$]) 337.12340. Characterization using ^{1}H NMR, ^{13}C and IR is also performed (see Figures).

1.2 Synthesis of *o*P^6(OH)$_2$

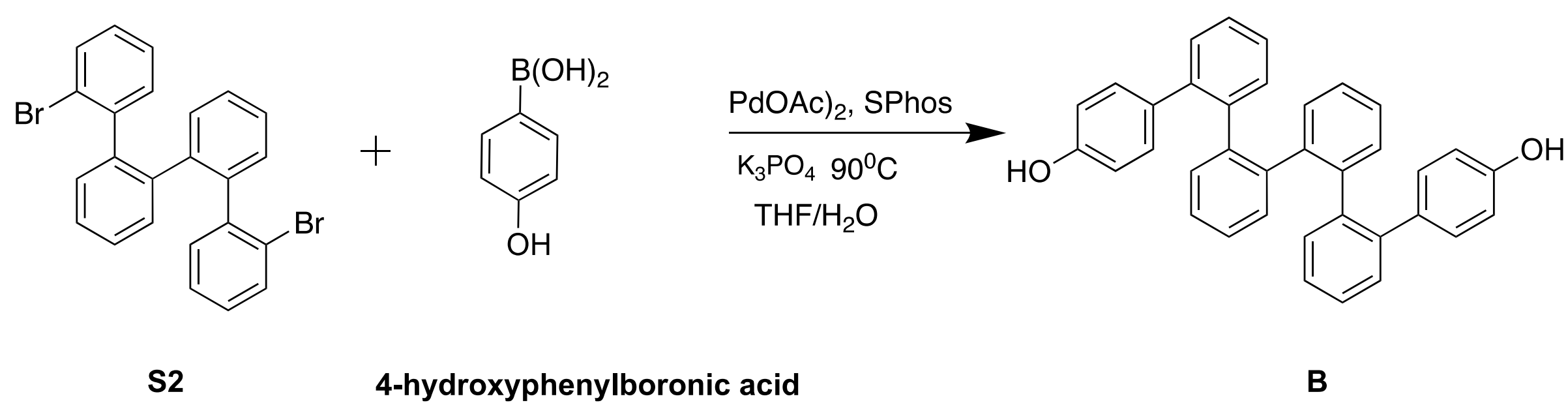

A Schlenk vacuum tube was charged with S2 (150 mg, 0.323 mmol, 1.0 eq), 4-hydroxyphenylboronic acid (200 mg, 1.453 mmol, 4.5 eq), palladium acetate (14.5 mg, 0.0646 mmol, 0.2 eq), SPhos (32 mg, 0.0775 mmol, 0.24 eq), and potassium phosphate (343 mg, 1.615 mmol, 5.0 eq) then evacuated and backfilled with argon (3×). Tetrahydrofuran (5 mL) and water (1.25 mL) were then added. The Schlenk tube was degassed by three freeze-pump-thaw cycles, backfilled with argon, then sealed and heated to 90 °C (silicone oil bath) for 24 h. The reaction was then cooled and diluted with ethyl acetate. The organic layer was washed with water (3×) and brine, then dried over $MgSO_4$, filtered, and concentrated under vacuum. The crude products when purified via flash chromatography (1:4 EtOAc:Hexanes) to give B as a white solid (75 mg, 47.3%) mp: 161.39 °C; HRMS (MALDI) calc'd for $C_{36}H_{25}O_2$ 489.1885, found ([M-H$^+$]) 489.186000.

1.3 Synthesis of 2-(propionic acid)yldodecyl trithiocarbonate (PADTC)

PADTC was synthesized according to the previously published procedures from the literature[2].

1.4 Synthesis of HY-(PADTC)$_2$

Hydroquinone (1 g, 0.0098 mol) was added to a 500 mL round-bottom flask equipped with a magnetic stirrer bar containing 4-dimethylaminopyridine (DMAP) (1.38 g, 0.01 mmol), PADTC (7.96 g, 0.02 mmol) and dichloromethane (DCM) (150 mL). The reaction solution was then cooled in an ice bath and EDC (5.28g, 0.03 mol) was added. The reaction mixture was capped and left to stir at room temperature for 24 h. The DCM solution was washed three times with 0.2 M HCl solution, twice with 0.5 M NaOH solution, once with water, and twice with saturated brine solution. The combined organic layer was dried over anhydrous Na_2SO_4 and concentrated under reduced pressure to give 6.3g (70%) of yellow solid. ^{1}H NMR was used to confirm the purified product (see Figure S12a).

1.5 Synthesis of *o*P^4-(PADTC)$_2$

Procedure in 1.4 above was followed except that *o*P^4(OH)$_2$ was used instead of hydroquinone. 73% of the product (yellow solid) was obtained. The compound was characterized using ^{1}H NMR (see Figure S12 b).

1.6 Synthesis of *o*P^6-(PADTC)$_2$

Procedure in 1.4 above was followed except that *o*P^6(OH)$_2$ was used instead of hydroquinone. 64% of the product (yellow solid) was obtained. The compound was characterized using ^{1}H NMR (see Figure S12c).

1.7 Polymer network synthesis (FMA-based networks)

1.7.1 Synthesis of HY-(PADTC)$_2$ and FMA-based DP 50 network

AIBN (0.0029 g, 1.8 x 10^{-5} mmol), HY-(PADTC)$_2$ (0.14g, 0.00018 mmol), ethyl acrylate (EA) (0.9 g, 0.009 mmol), furfuryl methacrylate (FMA) (0.15 g, 0.0009 mmol), and tetrahydrofuran (THF) (1.5 g) were added to a 25 mL round bottom flask and capped with a rubber septum. The resulting reaction mixture was purged with argon for 20 min to deoxygenate and then placed in an oil bath at 60 °C for 8 h. ^{1}H NMR was used to determine the conversion of monomers to be >75%. The crude product was precipitated in hexane to get rid of unreacted starting materials and dried under reduced pressure at 30 °C for 24 h. Dynamic DA crosslinking was done by addition of 0.5 mole equivalent of 1,1′-(methylene-di-4,1-phenyl-ene)bismaleimide (BMI) with respect to the mole of FMA present and sonicated for 30 min at room using DMF as the solvent. After mixing, the mixture of the copolymer and BMI was transferred into a dogbone-shaped teflon mold immediately. The network polymer was left to crosslink overnight at 40 °C and taken out of the mold to dry for 24 h. Further drying was done in the vacuum oven overnight and in the oven at 90 °C for 24 h. The network was allowed to cool down before characterization was done.

1.7.2 Synthesis of *o*P^4-(PADTC)$_2$ and FMA-based DP 50 network

AIBN (0.0029 g, 1.8 x 10^{-5} mmol), *o*P^4-$(PADTC)_2$ (0.18g, 0.00018 mmol), ethyl acrylate (EA) (0.9 g, 0.009 mmol), furfuryl methacrylate (FMA) (0.15 g, 0.0009 mmol), and tetrahydrofuran (THF) (1.5 g) were added to a 25 mL round bottom flask and capped with a rubber septum. The resulting reaction mixture was purged with argon for 20 min to deoxygenate and then placed in an oil bath at 60 °C for 8 h. ^1H NMR was used to determine the conversion of monomers to be >75%. The crude product was precipitated in hexane to get rid of unreacted starting materials and dried under reduced pressure at 30 °C for 24 h. Dynamic DA crosslinking was done by addition of 0.5 mole equivalent of 1,1′-(methylene-di-4,1-phenyl-ene)bismaleimide (BMI) with respect to the mole of FMA present and sonicated for 30 min at room using DMF as the solvent. After mixing, the mixture of the copolymer and BMI was transferred into a dogbone-shaped teflon mold immediately. The network polymer was left to crosslink overnight at 40 °C and taken out of the mold to dry for 24 h. Further drying was done in the vacuum oven overnight and in the oven at 90 °C for 24 h. The network was allowed to cool down before characterization was done.

1.7.3 Synthesis of *o*P^6-$(PADTC)_2$ and FMA-based DP 50 network

AIBN (0.0029 g, 1.8 x 10^{-5} mmol), *o*P^6-$(PADTC)_2$ (0.21g, 0.00018 mmol), EA (0.90 g, 0.009 mmol), furfuryl methacrylate (FMA) (0.15 g, 0.0009 mmol), and tetrahydrofuran (THF) (1.5 g) were added to a 25 mL round bottom flask and capped with a rubber septum. The resulting reaction mixture was purged with argon for 20 min to deoxygenate and then placed in an oil bath at 60 °C for 8 h. ^1H NMR was used to determine the conversion of monomers to be >75%. The crude product was precipitated in hexane to get rid of unreacted starting materials and dried under reduced pressure at 30 °C for 24 h. Dynamic DA crosslinking was done by addition of 0.5 mole equivalent of 1,1′-(methylene-di-4,1-phenyl-ene)bismaleimide (BMI) with respect to the mole of FMA present and sonicated for 30 min at room using DMF as the solvent. After mixing, the mixture of the copolymer and BMI was transferred into a dogbone-shaped teflon mold immediately. The network polymer was left to crosslink overnight at 40 °C and taken out of the mold to dry for 24 h. Further drying was done in the vacuum oven overnight and in the oven at 90 °C for 24 h. The network was allowed to cool down before characterization was done.

1.7.4 Synthesis of *o*P^4-$(PADTC)_2$ and FMA-based DP 100 network

AIBN (0.18 g, 0.00018 mmol), *o*P^4-$(PADTC)_2$ (0.14g, 0.00018 mmol), EA (1.8 g, 0.018 mmol), furfuryl methacrylate (FMA) (0.15 g, 0.0009 mmol), and THF (3 g) were added to a 25 mL round bottom flask and capped with a rubber septum. The resulting reaction mixture was purged with argon for 20 min to deoxygenate and then placed in an oil bath at 60 °C for 8 h. ^1H NMR was used to determine the conversion of monomers to be >75%. The crude product was precipitated in hexane to get rid of unreacted starting materials and dried under reduced pressure at 30 °C for 24 h. Dynamic DA crosslinking was done by addition of 0.5 mole equivalent of 1,1′-(methylene-di-4,1-phenyl-ene)bismaleimide (BMI) with respect to the mole of FMA present and sonicated for 30 min at room using DMF as the solvent. After mixing, the mixture of the copolymer and BMI was transferred into a dogbone-shaped teflon mold immediately. The network polymer was left to crosslink overnight at 40 °C and taken out of the mold to dry for 24 h. Further drying was done in the vacuum oven overnight and in the oven at 90 °C for 24 h. The network was allowed to cool down before characterization was done.

1.8 Polymer network synthesis (EGDMA-based networks)

1.8.1 Synthesis of EGDMA-based network

Phenylbis(2,4,6-trimethylbenzoyl) phosphineoxide (BAPO) (0.42 g, 0.001 mmol), HY-$(PADTC)_2$ (0.16g, 0.0002 mmol), EA (1.01 g, 0.01 mmol), ethylene glycol dimethacrylate (EGDMA) (0.2 g, 0.001 mmol), and dimethylformamide (DMF) (1.5 g) were added to a 10 mL vial and sonicated at room temperature until all the mixtures are fully dissolved and homogenous. After mixing, the mixture was transferred into a dogbone-shaped teflon mold immediately. The mixture in the mold was placed under a UV lamp of 360 nm and was left to polymerize and crosslink for 3 h at room temperature. The crosslinked networks were taken out of the mold to dry for 24 h. Further drying was done in the vacuum oven overnight and in the oven at 90 °C for 24 h. The network was allowed to cool down before characterization was done.

1.8.2 Synthesis of oP^4-PADTC and EGDMA-based network

BAPO (0.42 g, 0.001 mmol), oP^4-$(PADTC)_2$ (0.20g, 0.0002 mmol), EA (1.01 g, 0.01 mmol), EGDMA (0.2 g, 0.001 mmol), and DMF (1.5 g) were added to a 10 mL vial and sonicated at room temperature until all the mixtures are fully dissolved and homogenous. After mixing, the mixture was transferred into a dogbone-shaped teflon mold immediately. The mixture in the mold was placed under a UV lamp of 360 nm and was left to polymerize and crosslink for 3 h at room temperature. The crosslinked networks were taken out of the mold to dry for 24 h. Further drying was done in the vacuum oven overnight and in the oven at 90 °C for 24 h. The network was allowed to cool down before characterization was done.

1.9 Self-Healing Experiment

Two pristine polymer samples were used for the self-healing experiment. A pristine polymer material sample was cut into two pieces using a sharp razor blade while the other pristine polymer sample was left uncut. The two pieces cut samples were then put in close proximity together such that their fractured ends were in contact. A slight pressure was applied as shown in S3 (the purple arrows) to further ensure that cut samples stayed in contact before healing in the oven. Next, both pristine (uncut) and cut samples were heated at 90 °C for 24 h to ensure that uncut and healed polymer samples undergo similar condition for fair comparison. After oven healing, the samples were dried at room temperature for another 24 h before tensile experiment was carried out on the samples.

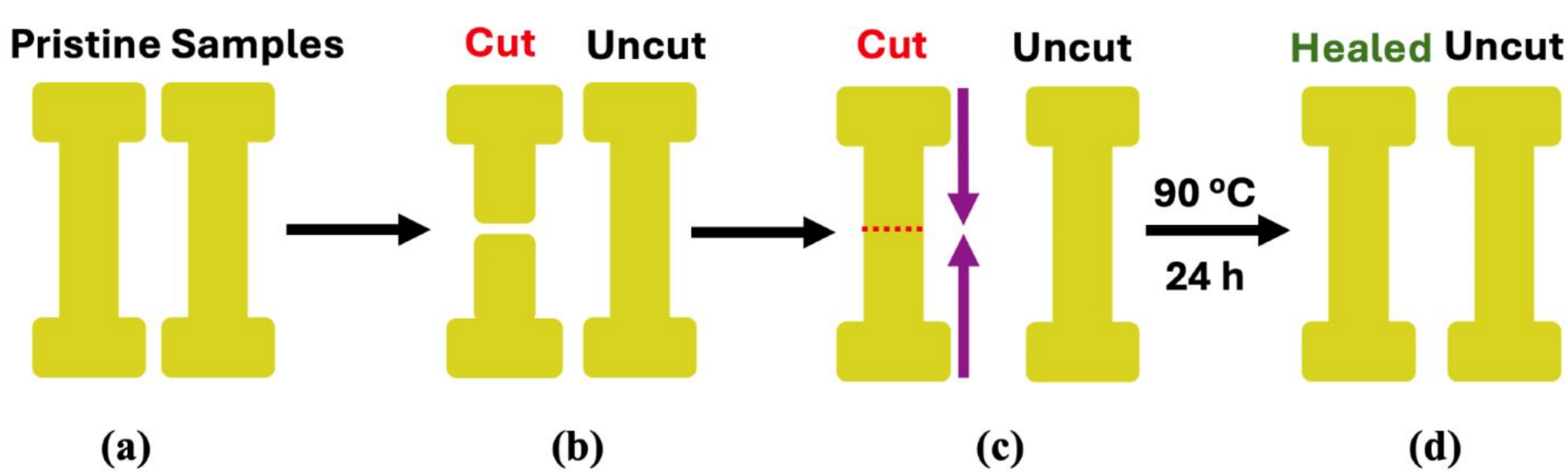

Fig. S1: Typical demonstration of self-healing experiment in this study. (a) Two pristine samples crosslinked with either dynamic DA or non-dynamic EGDMA. (b) A pristine sample was cut using

a sharp razor blade while the other pristine sample was left uncut. (c) Cut ends of the samples were placed close together to ensure fractured ends of cut samples were in contact and then put in the oven at 90 °C for 24 h (d) Healed and pristine samples were taken out of the oven and dried at room temperature.

Tensile Test Methods

Tensile testing was performed using an Instron 3344 Universal Testing System equipped with a 100 N load cell. All materials were tested at room temperature. Strain to break testing was performed at 3.6 mm min^{-1}. Cyclic loading/unloading was performed at extension rates of 3 mm min^{-1}, for three cycles of loading/unloading, with a maximum elongation of 30% of the original strain of the sample. Specimens were dogbone-shaped materials with typical length of ~15-20 mm, thickness of ~0.5-0.8 mm, and a cross-sectional area of ~2-4 mm^2. Hysteresis, Ξ, was evaluated as follows:

$$\Xi(\%) = \frac{\int_0^{\varepsilon_{30\%-max}} \sigma_{Load}(\varepsilon)d\varepsilon + \int_{\varepsilon_{30\%-max}}^{0} \sigma_{UnLoad}(\varepsilon)d\varepsilon}{\int_0^{\varepsilon_{30\%-max}} \sigma_{Load}(\varepsilon)d\varepsilon} \times 100 \qquad (S1)$$

where $\sigma_{Load}(\varepsilon)$ is the stress at a strain of ε in the loading branch, $\sigma_{UnLoad}(\varepsilon)$ is the stress at a strain of ε in the unloading branch and $\varepsilon_{30\%-max}$ is the strain that is 30% of the mean strain at break for that material.

Differential Scanning Calorimetry (DSC)

DSC was performed using a TA Instruments Q2000 DSC. The instrument was operated at a range of -60 °C to 150 °C, with a ramp rate of 10 °C min^{-1}. T_g was derived from the data plot of the second of two heat-cool-heat cycles run on each material sample.

Dynamic mechanical Analysis (DMA)

Temperature sweep DMA was performed on a TA Instruments Q800 equipped with a tension clamp. Analysis was performed under isothermal temperature/frequency sweep conditions, with a frequency range of 0.01 to 100 Hz at 35 °C. Under the testing conditions, material samples were held at temperature for three minutes prior to testing to equilibrate. Data was collected at a strain rate of 0.3%, with a preload force of 0.01 N.

Supplemental Data

Table S1: Self-healing properties of the non-dynamic crosslinked *o*P4-EGDMA and dynamic crosslinked *o*P4-FMA synthesized polymer networks.

Material	Target DP	σ_{peak} (kPa)	ε_{break} (mm/mm)
oP4-EGDMA (before healing)	50	960±40	1.3±0.1
oP4-EGDMA (after healing)	50	100±40	0.17±0.05
oP4-FMA (before healing)	50	1300±1	1.40±0.01
oP4-FMA (after healing)	50	1140±70	1.0±0.2

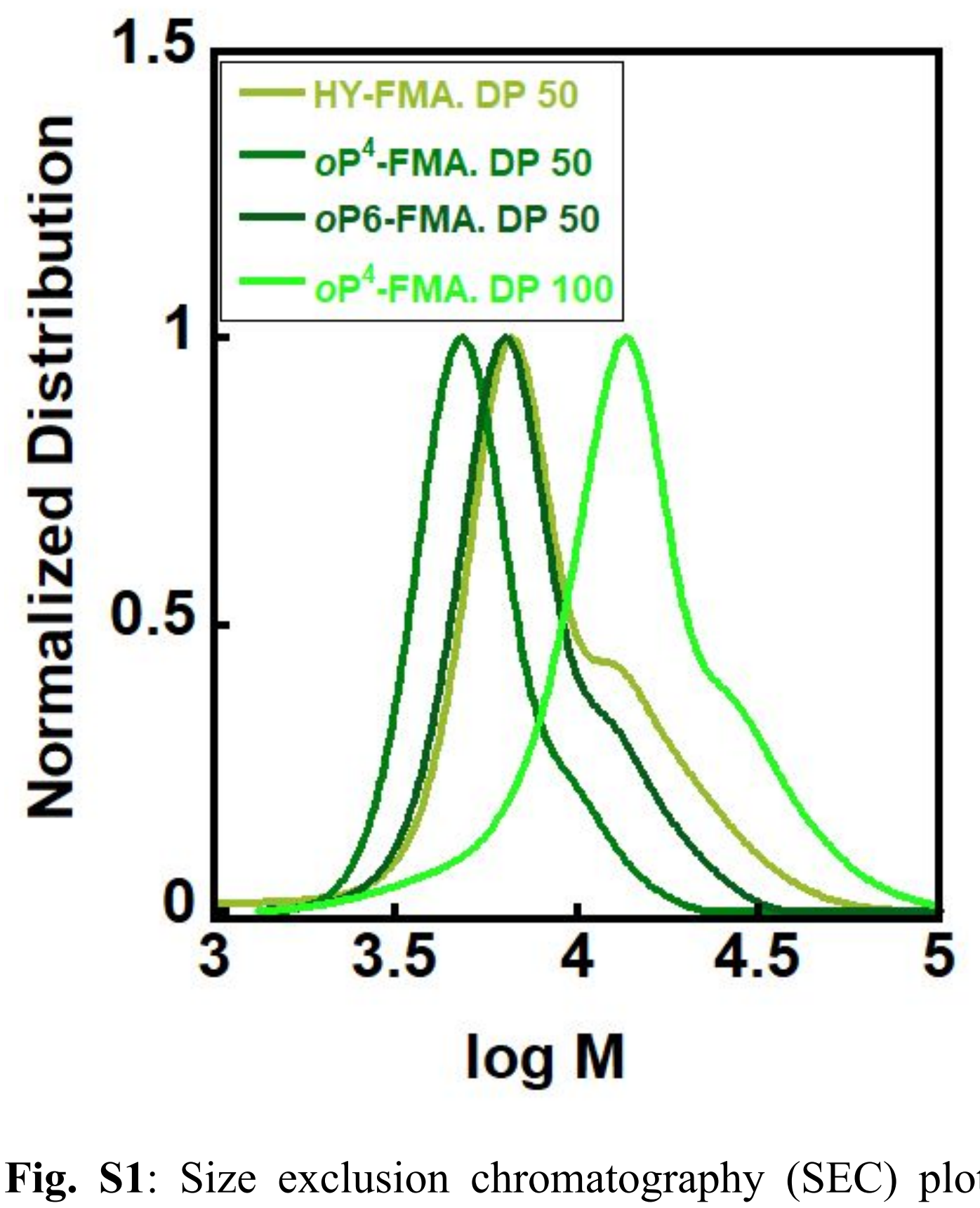

Fig. S1: Size exclusion chromatography (SEC) plot for synthesized networks with aromatic foldamer chain transfer agents.

Table S2: Molecular weight and conversion data for poly(EA/FMA) copolymers.

Material	Target DP	Conversion FMA	Conversion EA	M_n	Đ
HY-FMA	50	>90%	86%	7200	1.44
oP[4]-FMA	50	>90%	78%	4900	1.16
oP[6]-FMA	50	>90%	78%	6600	1.25
oP[4]-FMA	100	>90%	77%	12,000	1.45

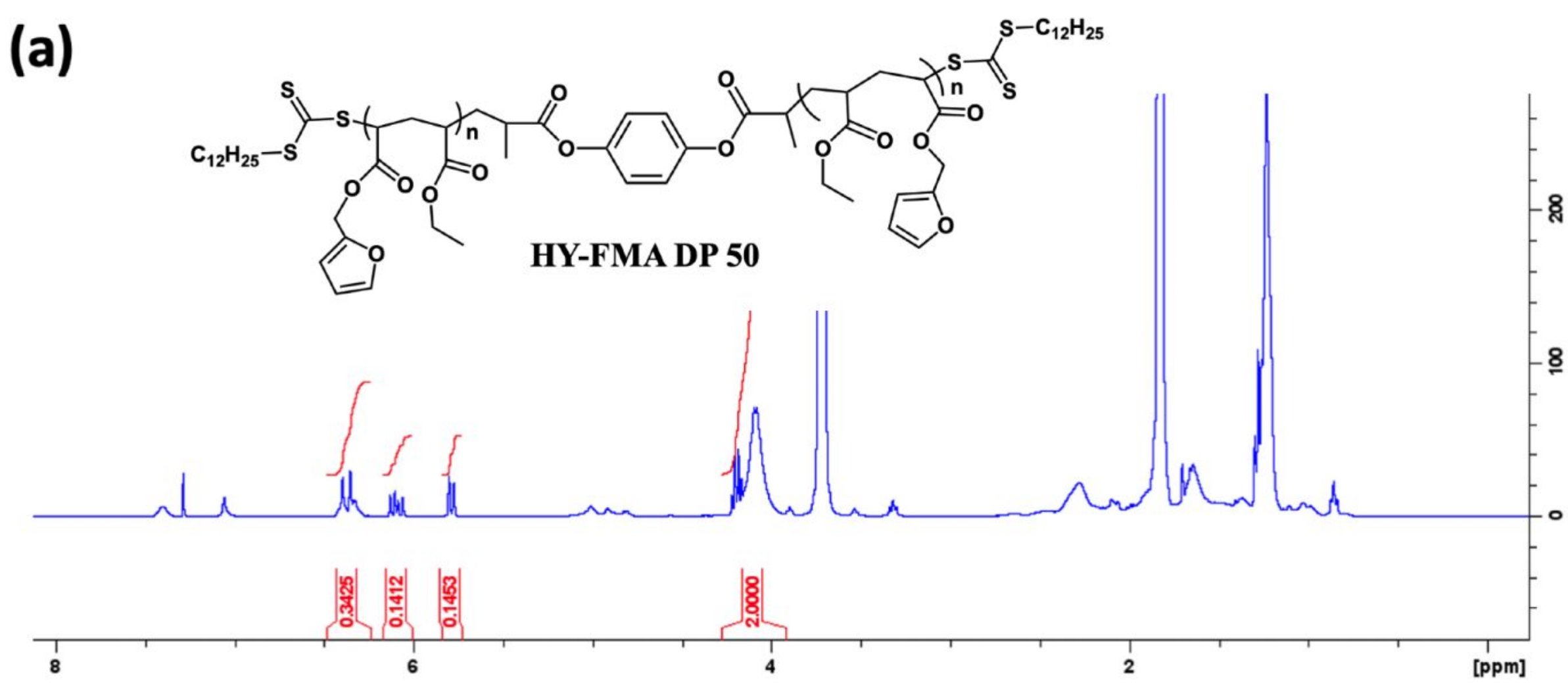

(b)

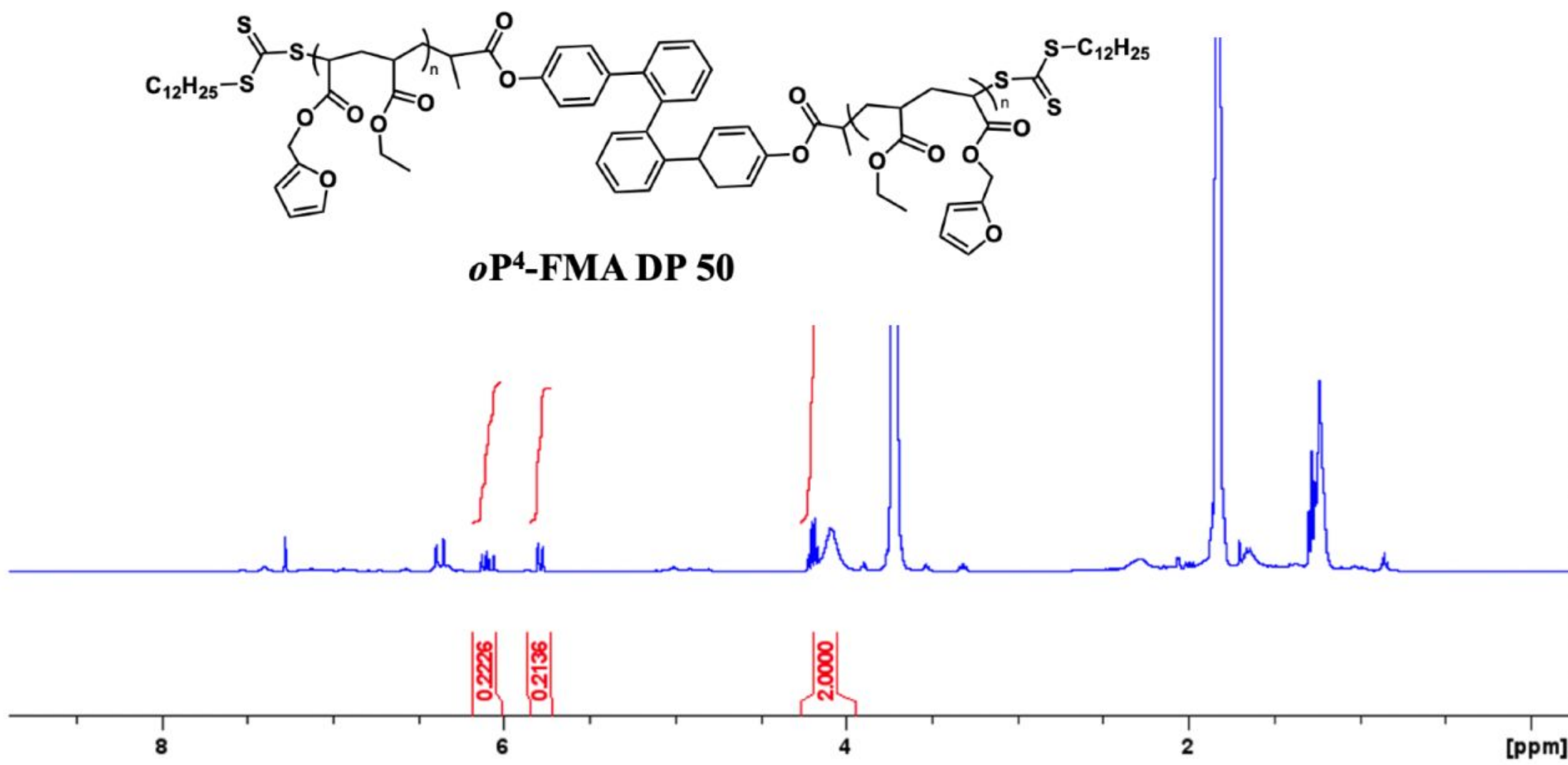

oP4-FMA DP 50
0.2226
0.2136
2.0000
[ppm]

(c)

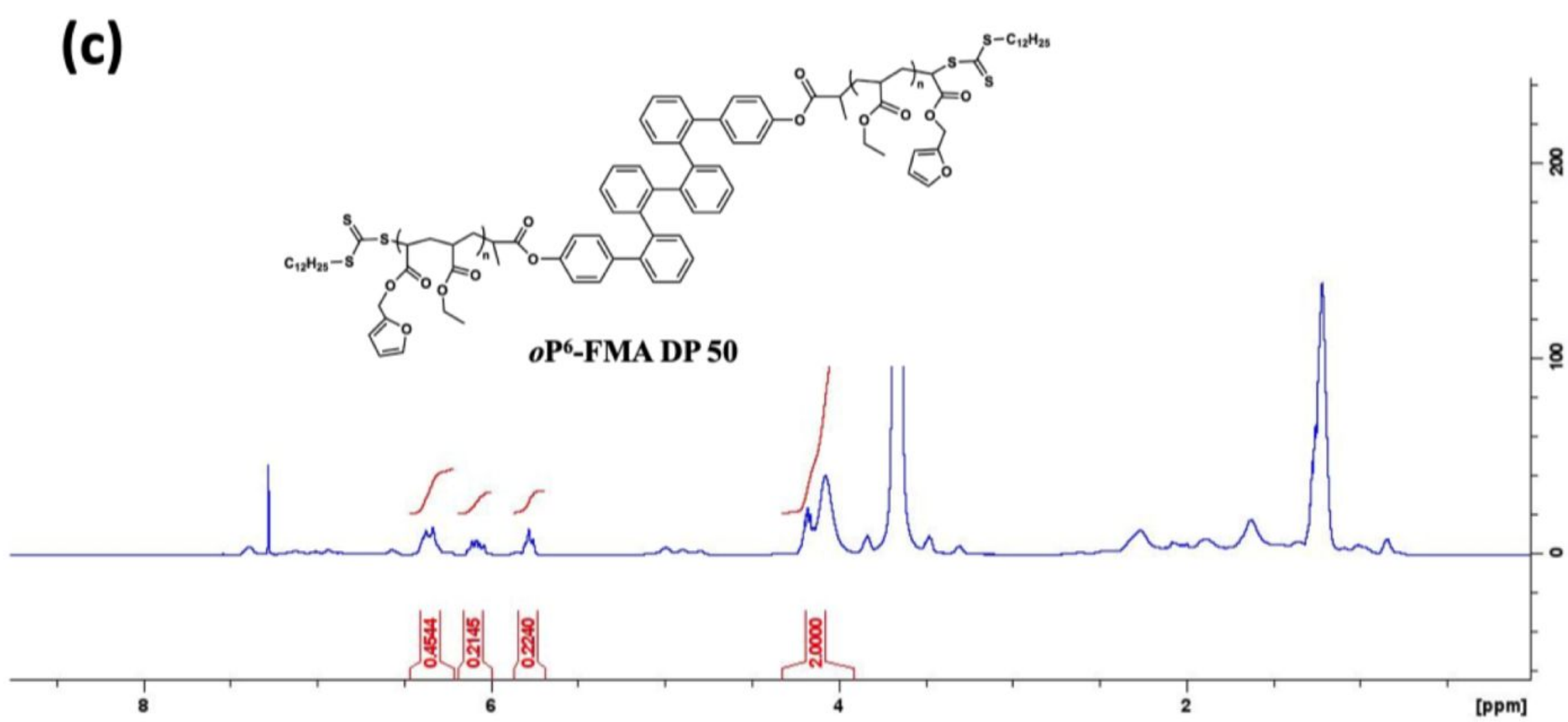

oP6-FMA DP 50
0.4544
0.2145
0.2240
2.0000
[ppm]

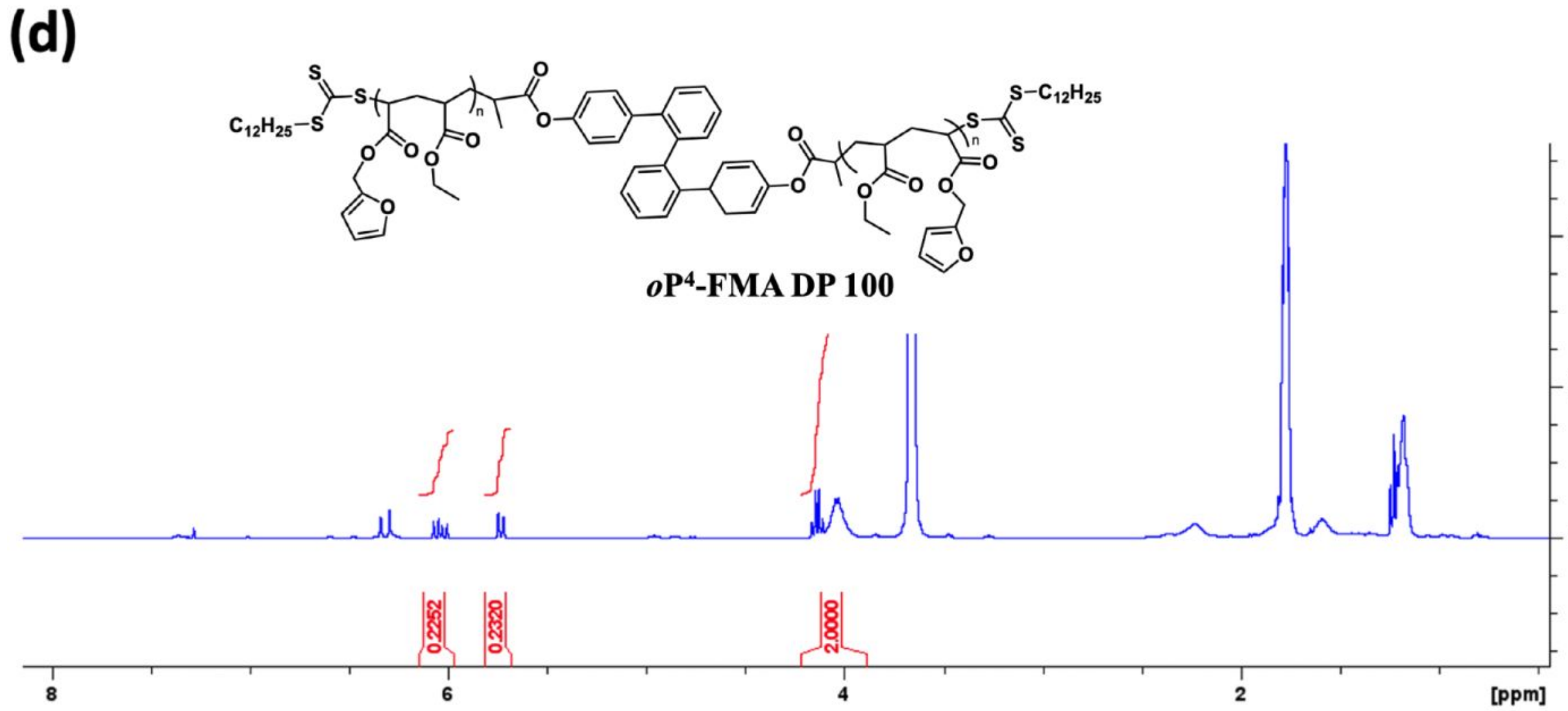

Fig S2: ^{1}H NMR spectra of the synthesized polymers for DP 50 (a) HY-FMA, (b) *o*P[4]-FMA, (c) *o*P[6]-FMA and (d) DP 100 *o*P[4]-FMA

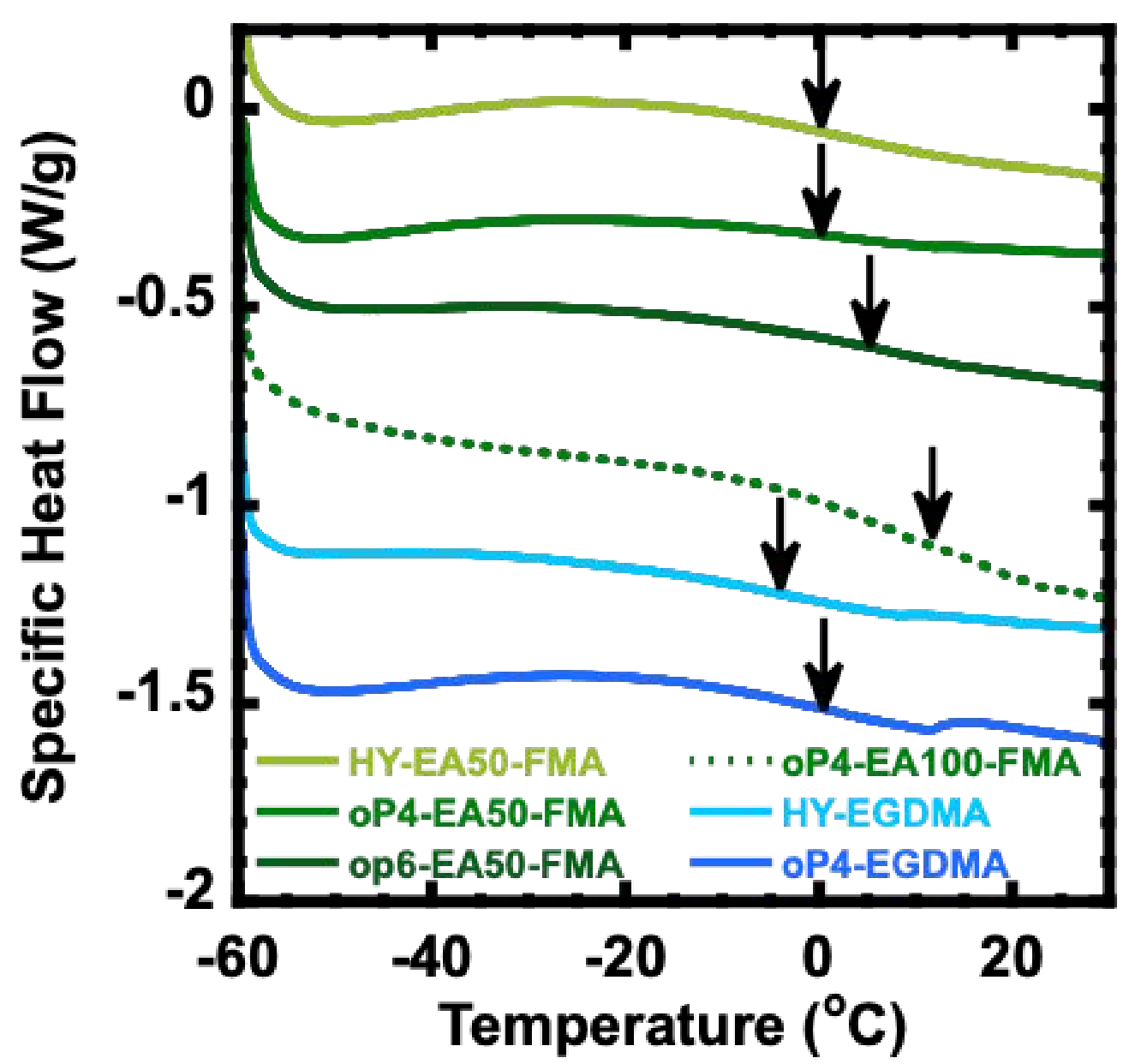

Fig S3: Differential scanning calorimetry (DSC) *Tg* plot of the networks. An offset of specific heat is given to facilitate differentiation of curves, and arrow shows the glass transition temperature (inflection point).

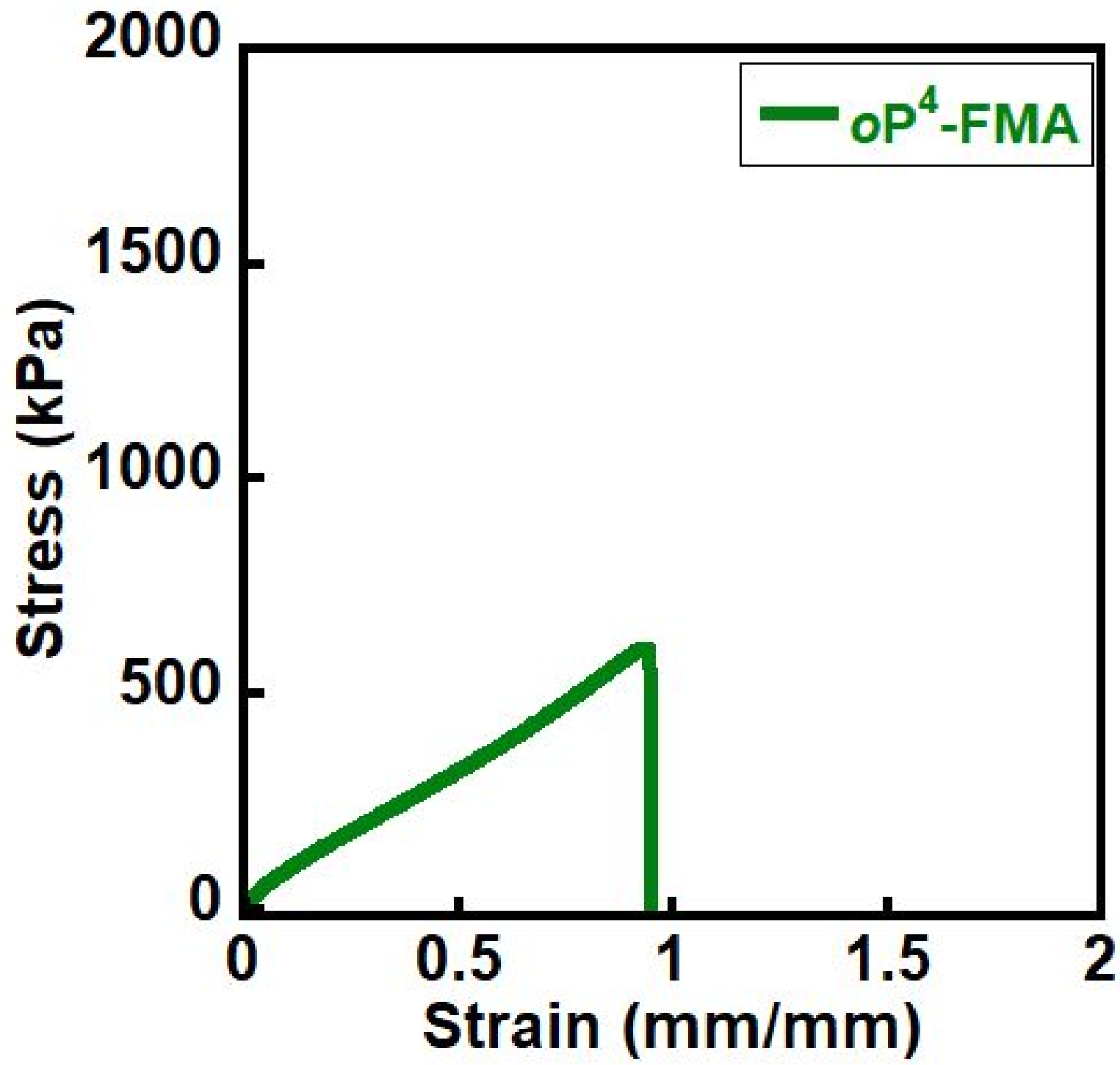

Fig S4: Tensile plot for DP 100 *o*P^4-FMA

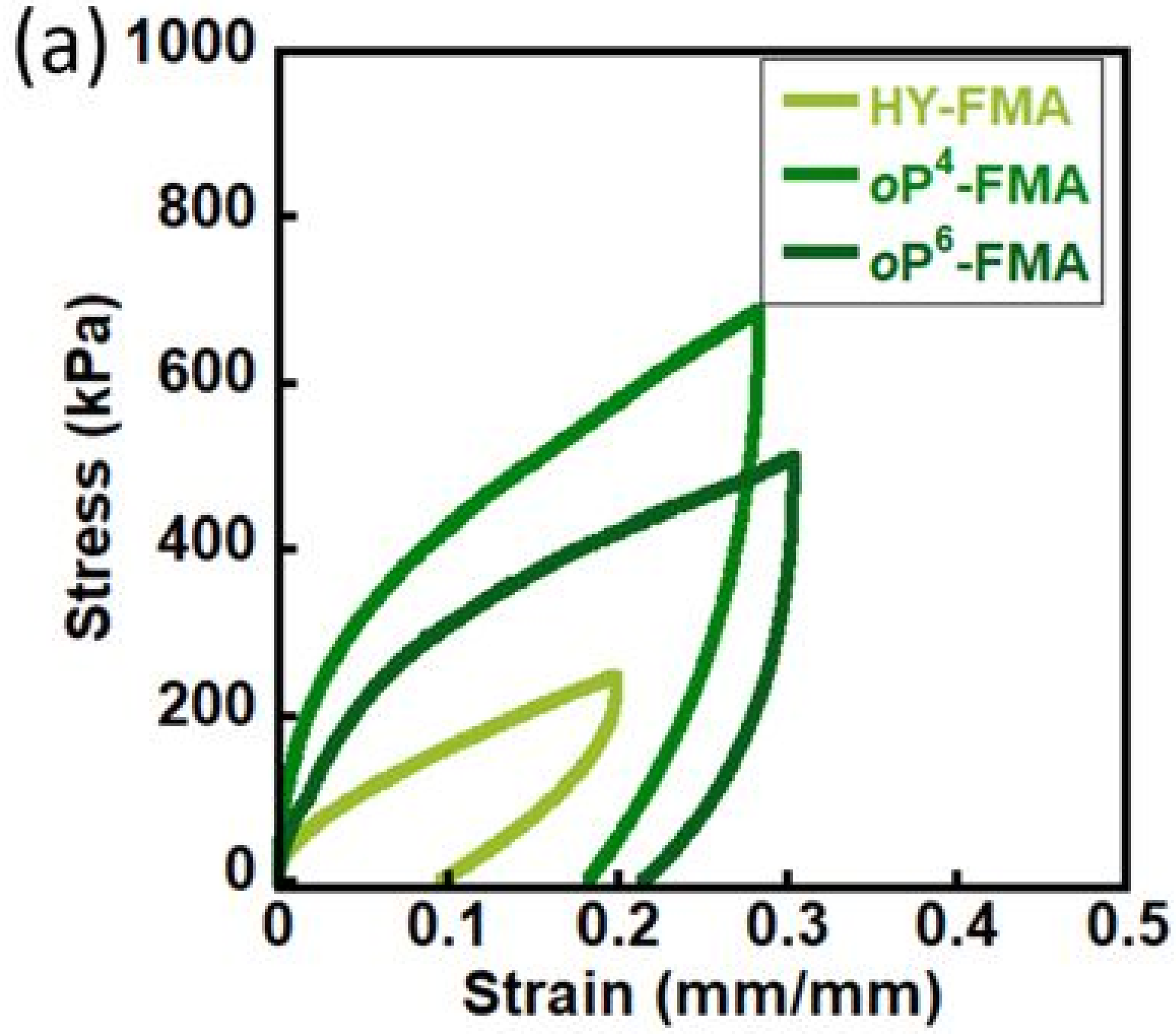

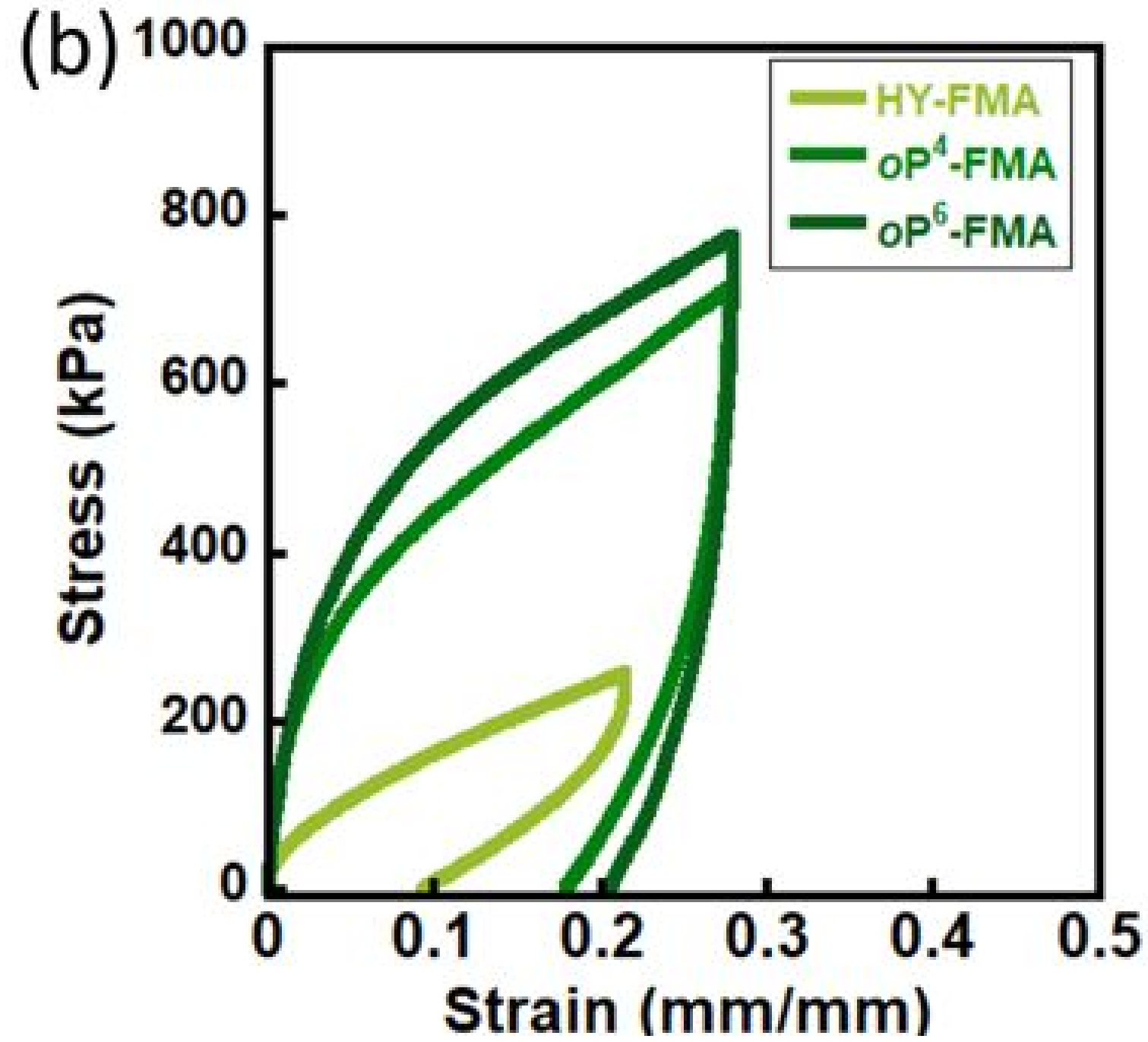

Fig. S5a-b: The loading–unloading cyclic curve for networks synthesized with aromatic foldamer RAFT CTAs (*o*P^4 and *o*P^6), the control (hydroquinone) and crosslinked with dynamic DA adduct are shown for 1 h and 2 h (of rest at 0 strain) respectively

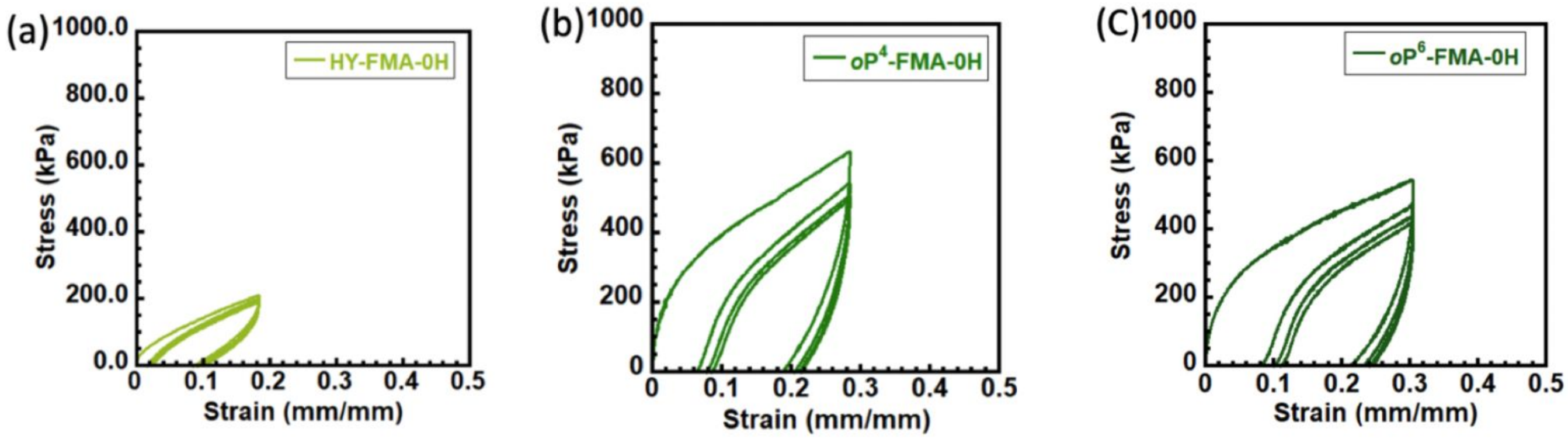

Fig S6: Four repeated cycles without rest interval between cycles for aromatic foldamer RAFT CTAs (*o*P^4 and *o*P^6), the control (hydroquinone) and crosslinked with dynamic DA adduct

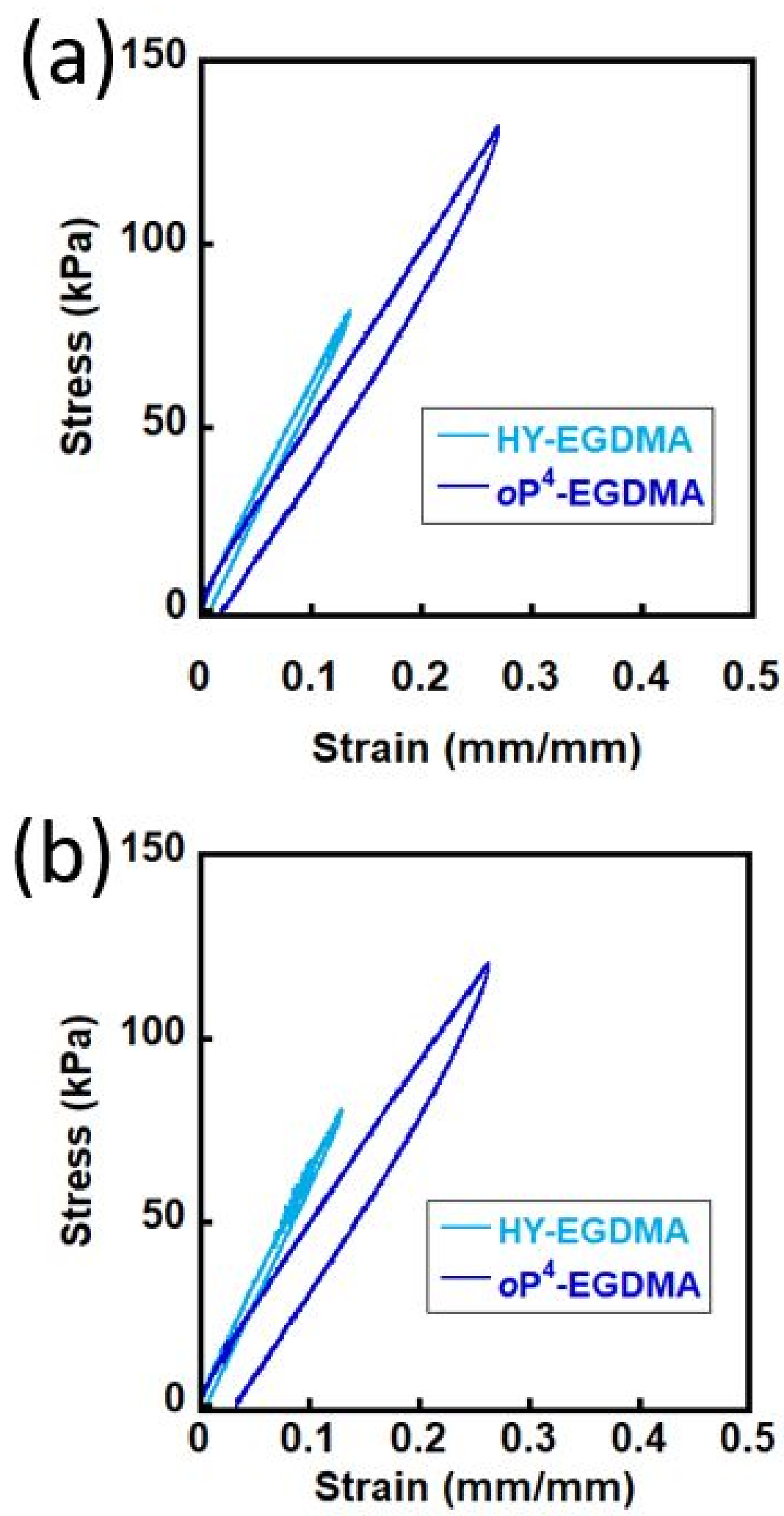

Fig S7a-b: The loading–unloading cyclic curve for networks synthesized with aromatic foldamer RAFT CTAs *o*P^4 and, the control (hydroquinone) crosslinked with non-dynamic EGDMA adduct are shown for 1 h and 2 h (of rest at 0 strain) respectively

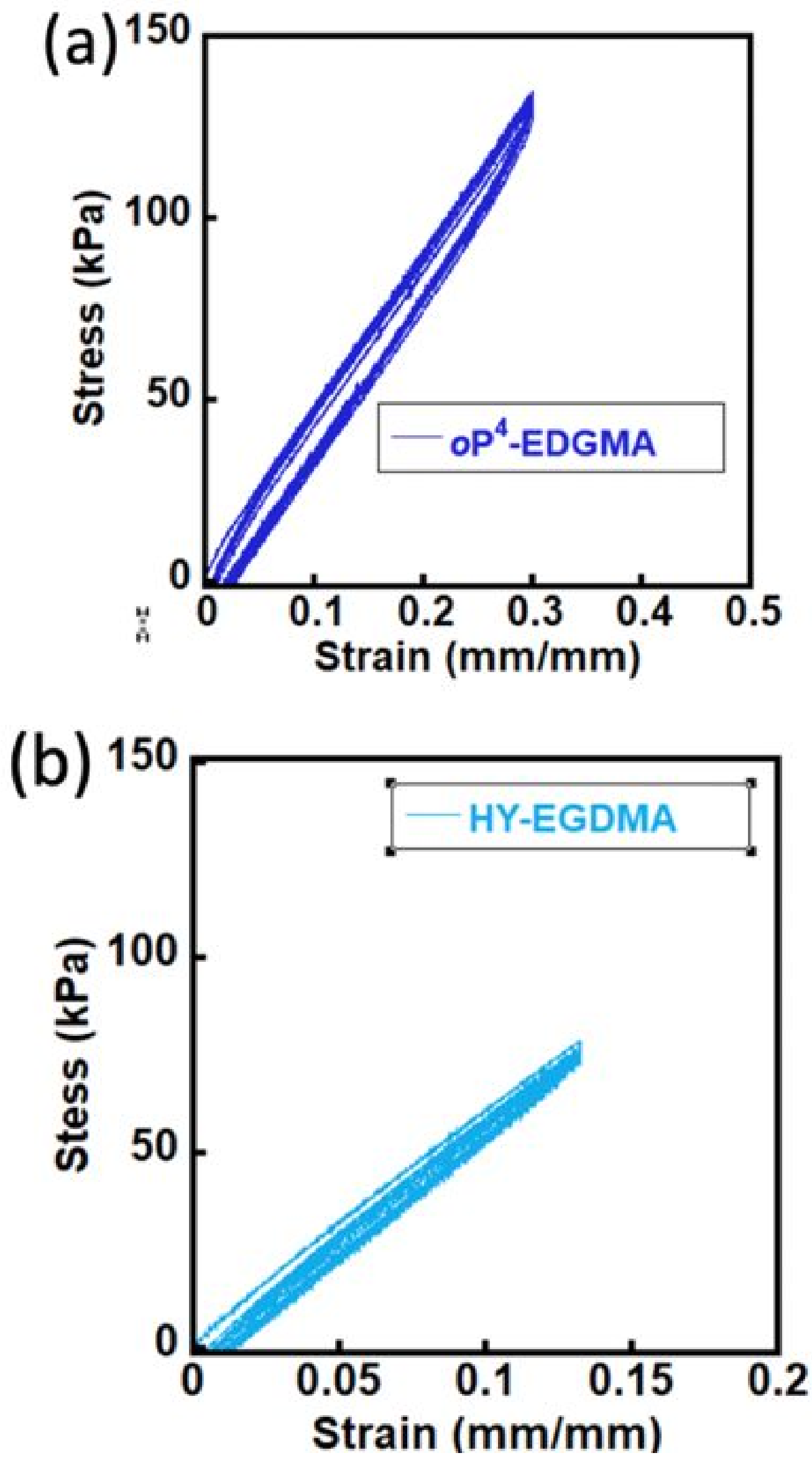

Fig S8: Four repeated cycles without rest interval between cycles for aromatic foldamer RAFT CTAs (a) *o*P^4 and the (b) control (hydroquinone) crosslinked with non-dynamic EGDMA

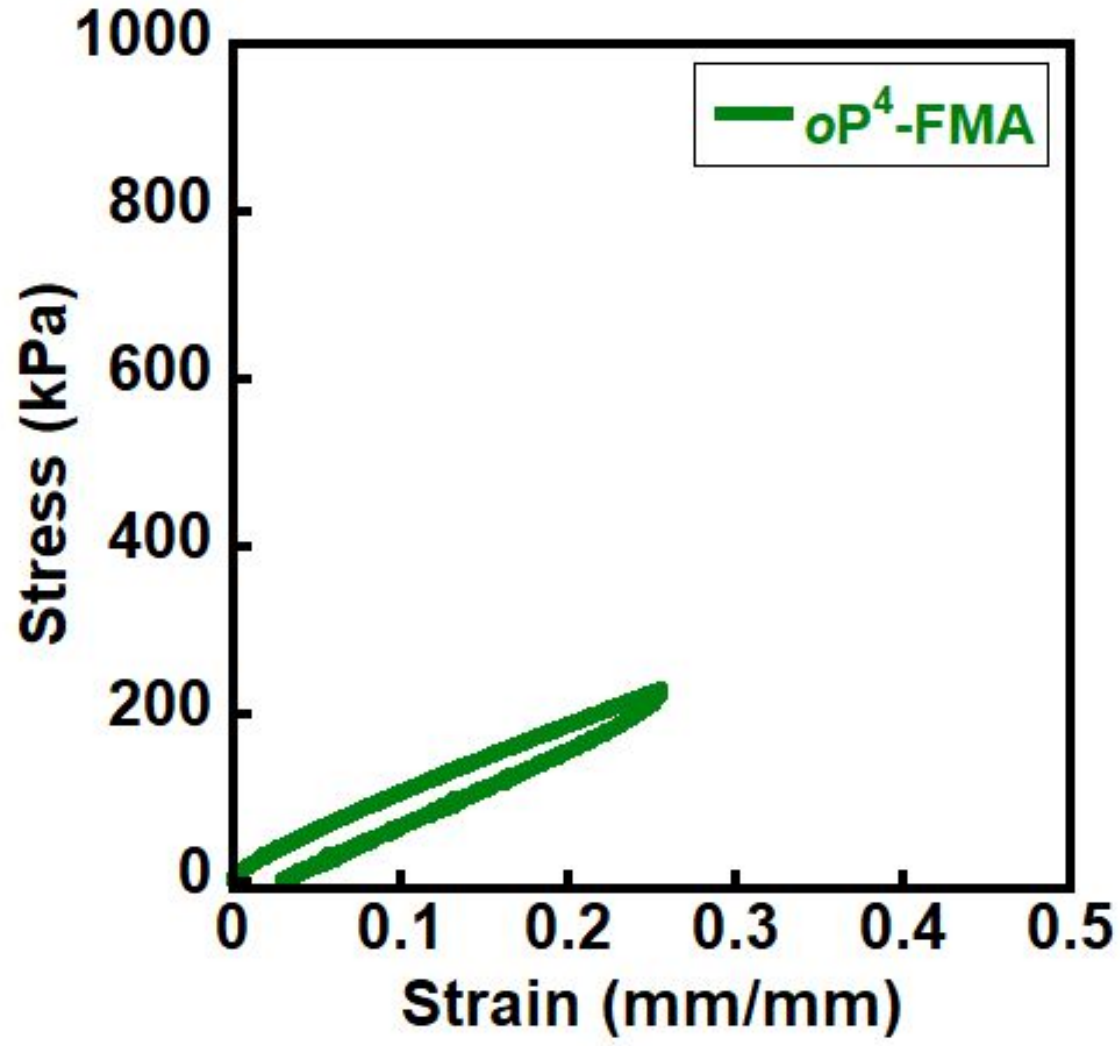

Fig S9: Cyclic tensile test on network synthesized from *o*P^4-CTA, DA adduct crosslinked but with DP of 100

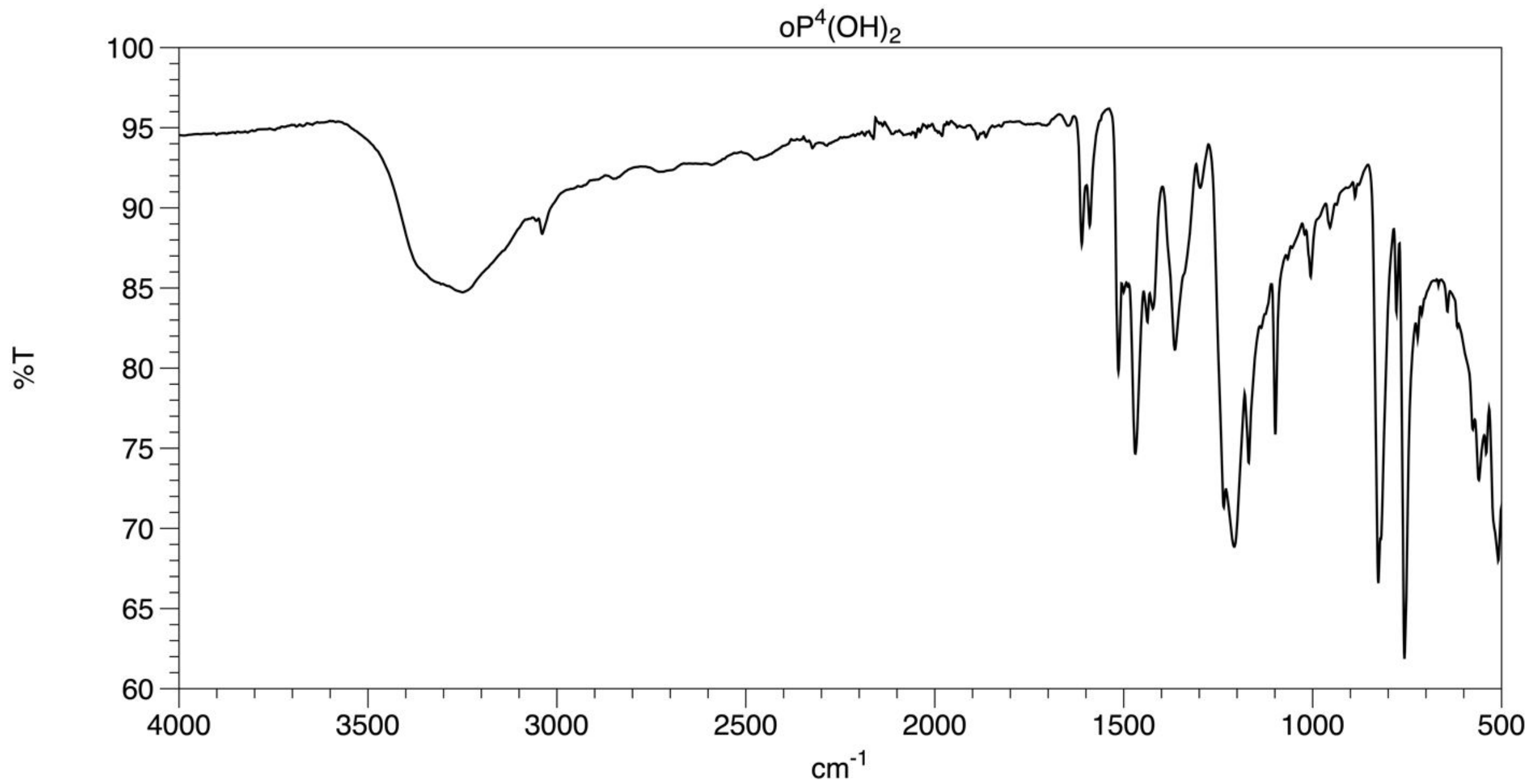

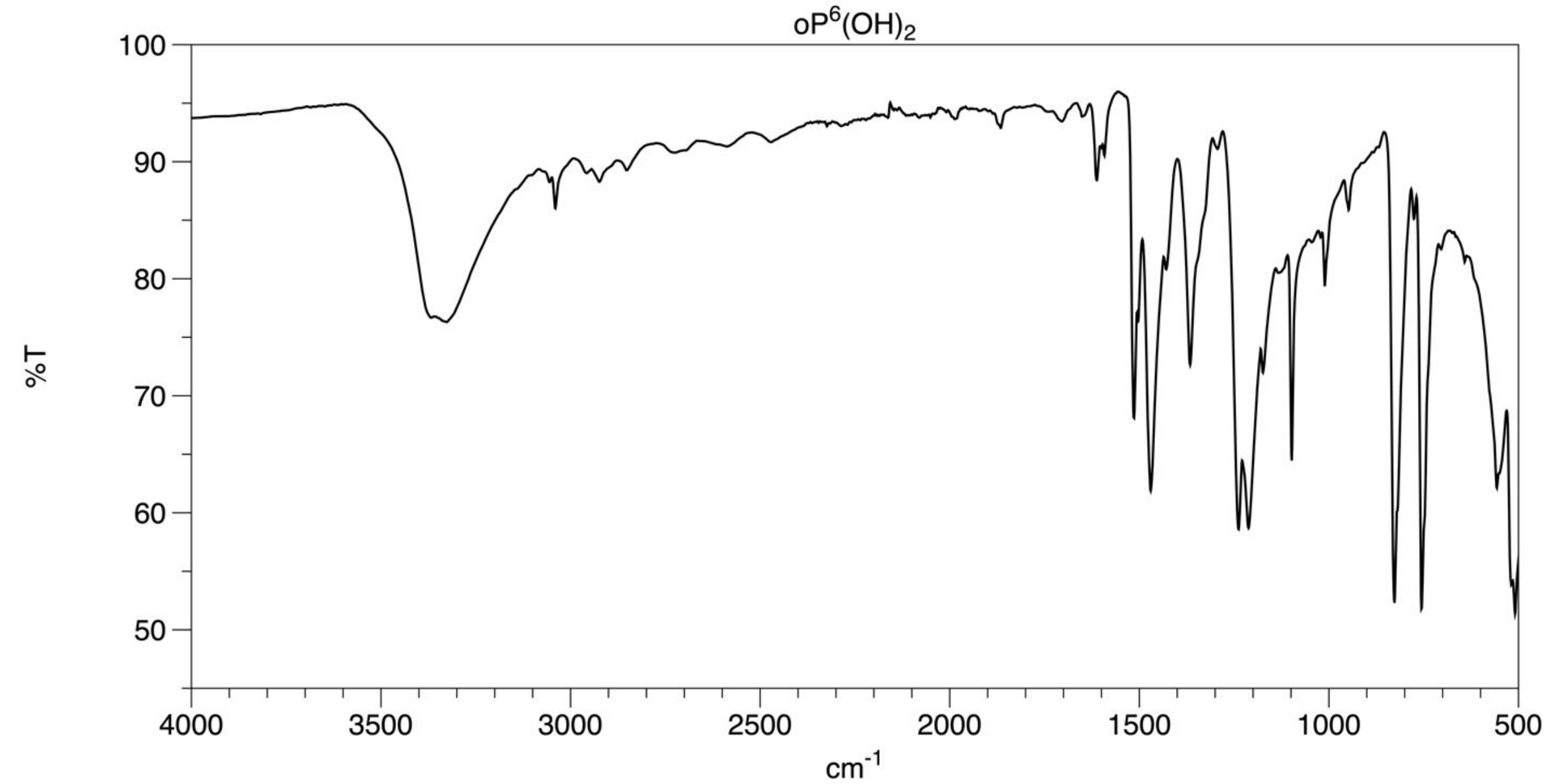

Fig S10: IR spectra for oP^4(OH)$_2$ and oP^6(OH)$_2$

(a)

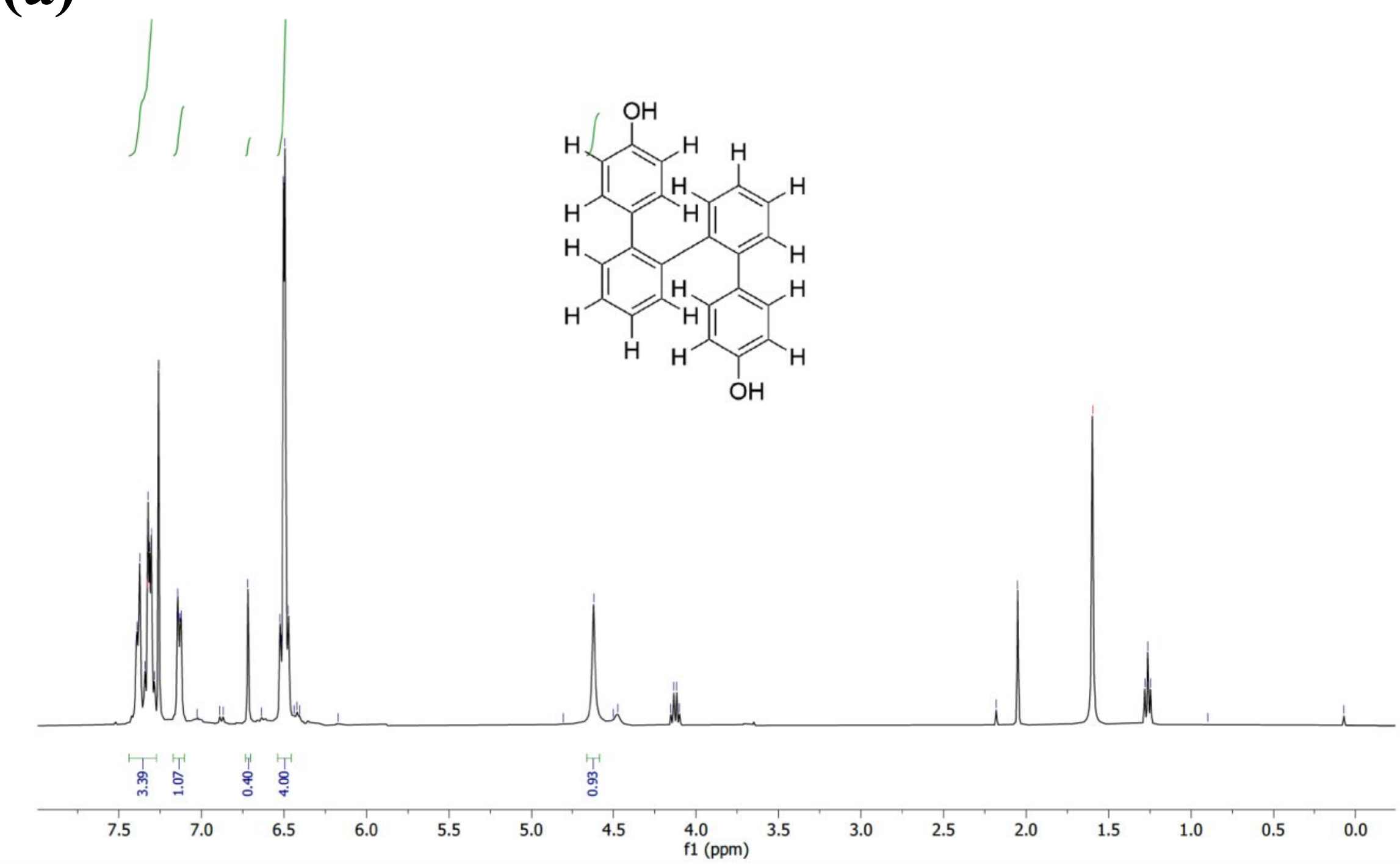

(b)

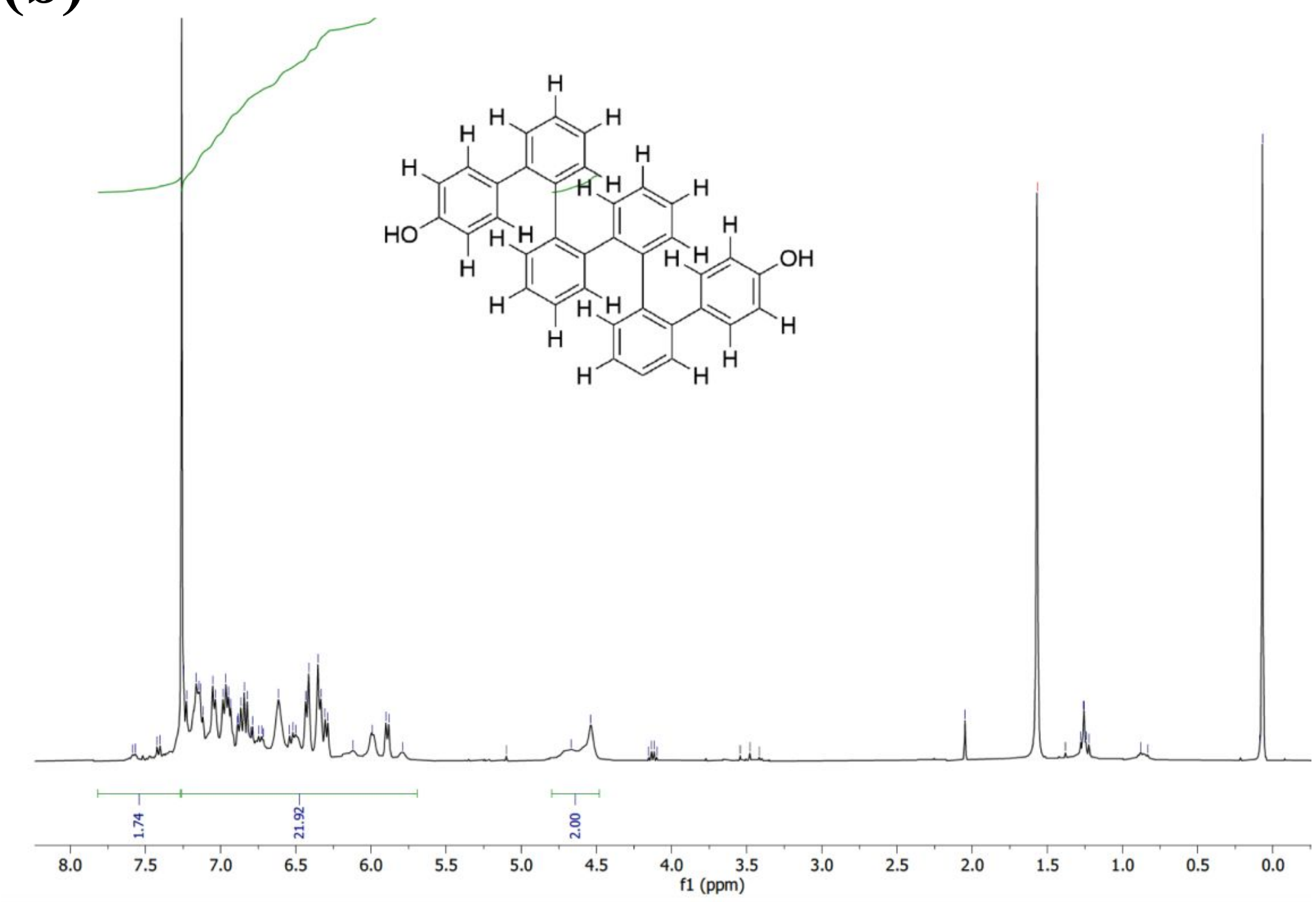

Fig S11: ^{1}H NMR spectra (400 MHz, $CDCl_3$) of (a) *o*$P^4(OH)_2$ and (b) *o*$P^6(OH)_2$

(a)

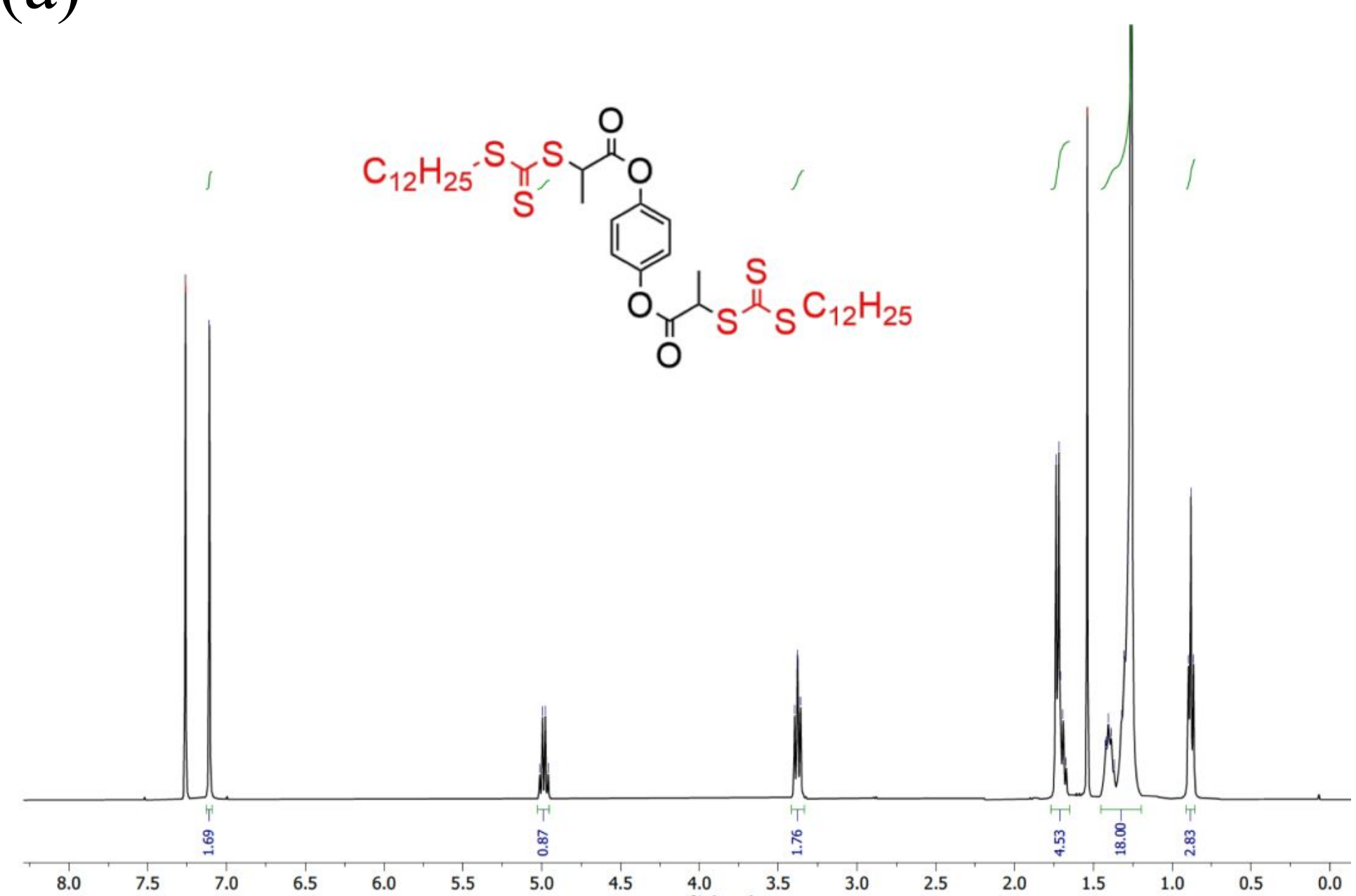

(b)

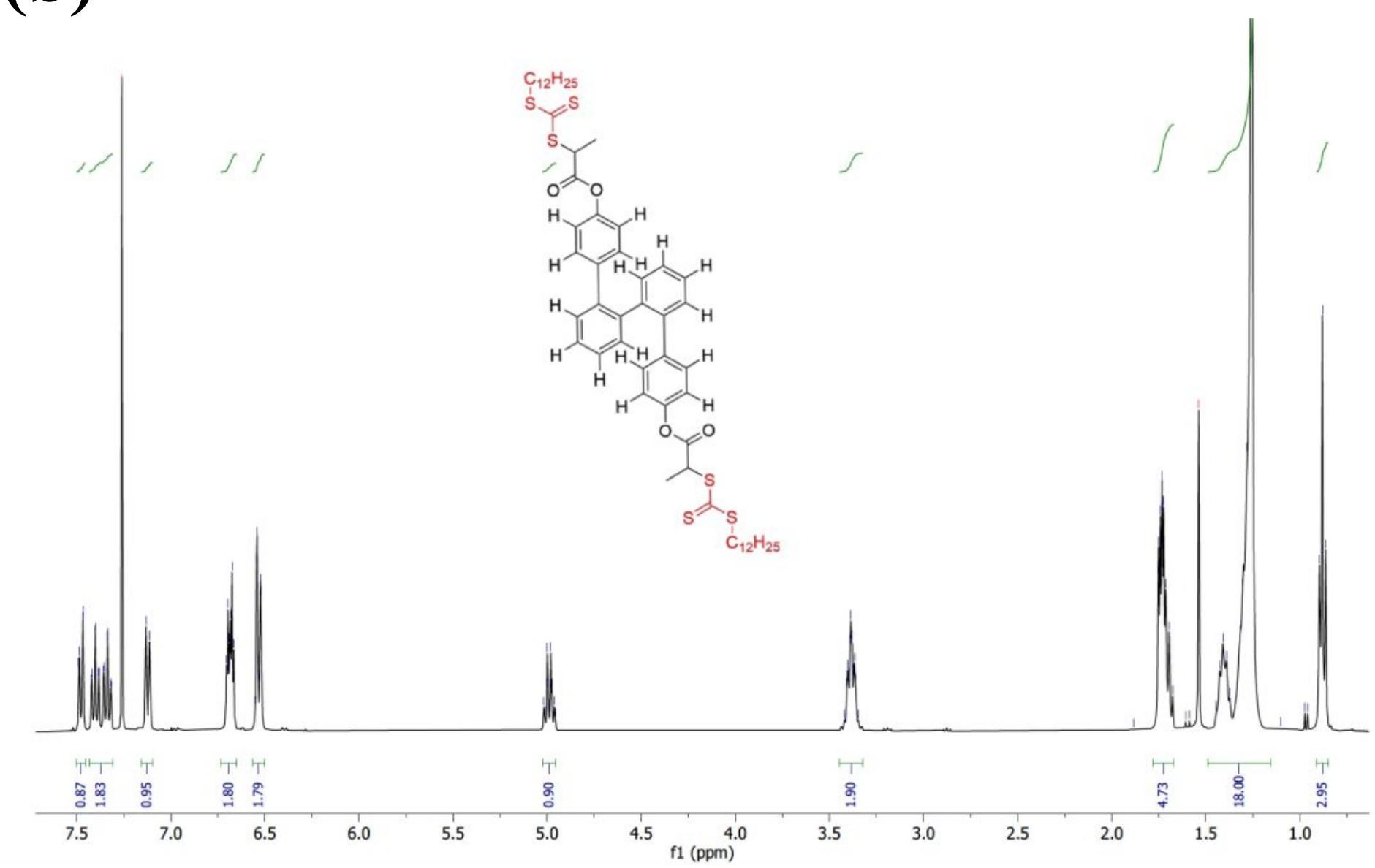

(c)

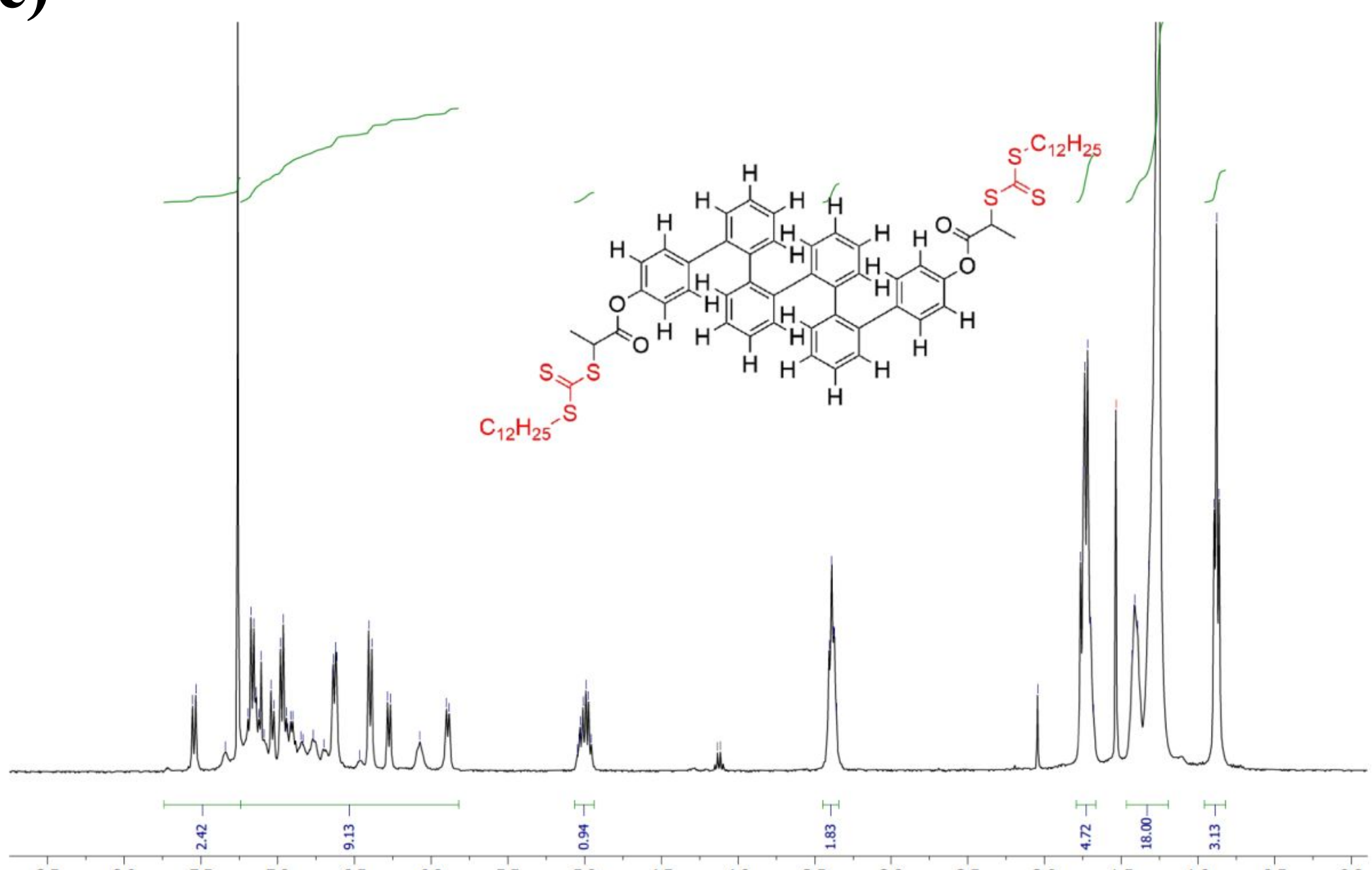

Fig S12: ^{1}H NMR spectra (400 MHz, $CDCl_3$) of (a) $HY(PADTC)_2$ (b) $oP^4(PADTC)_2$ and (c) $oP^6(PADTC)_2$.

(a)

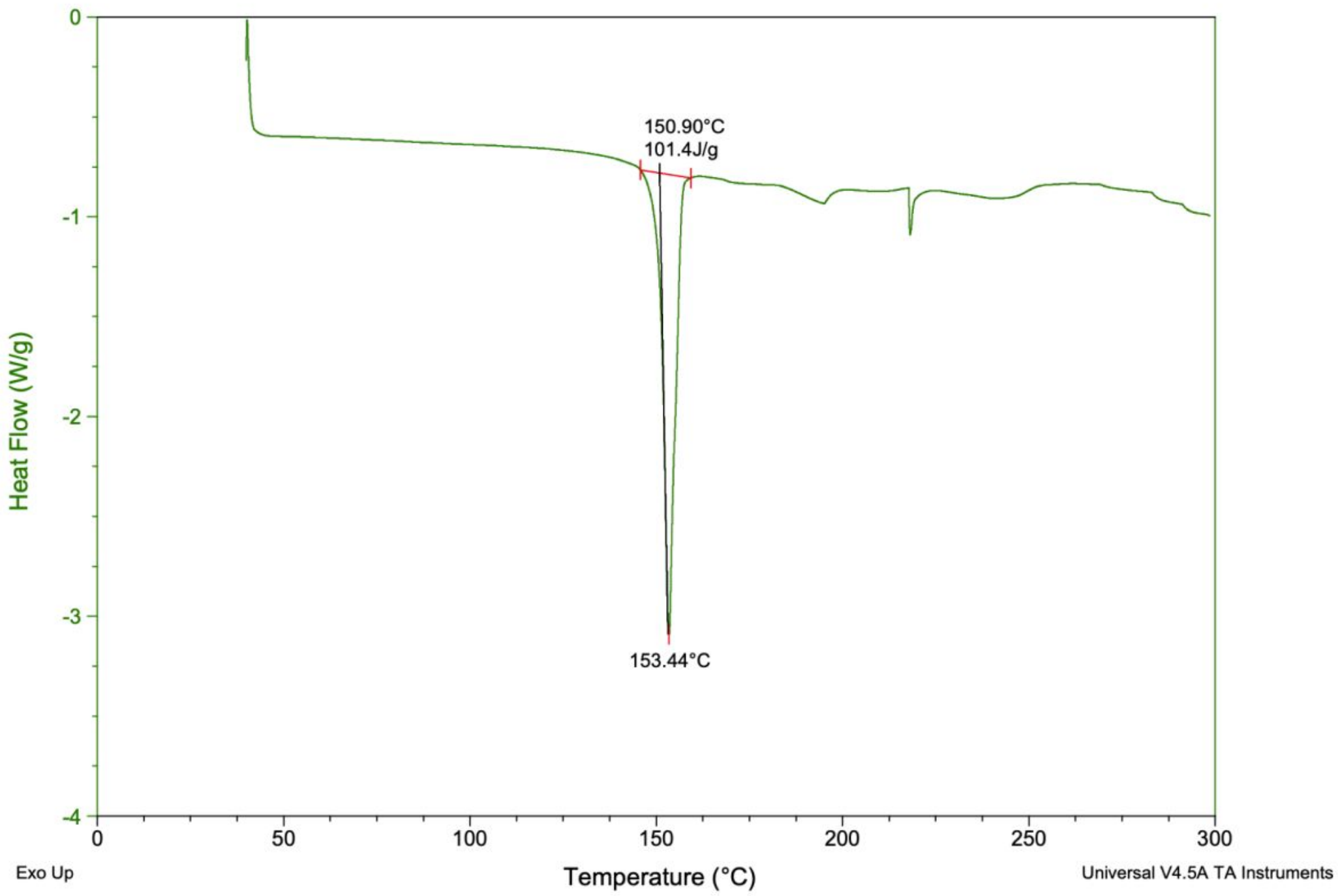

(b)

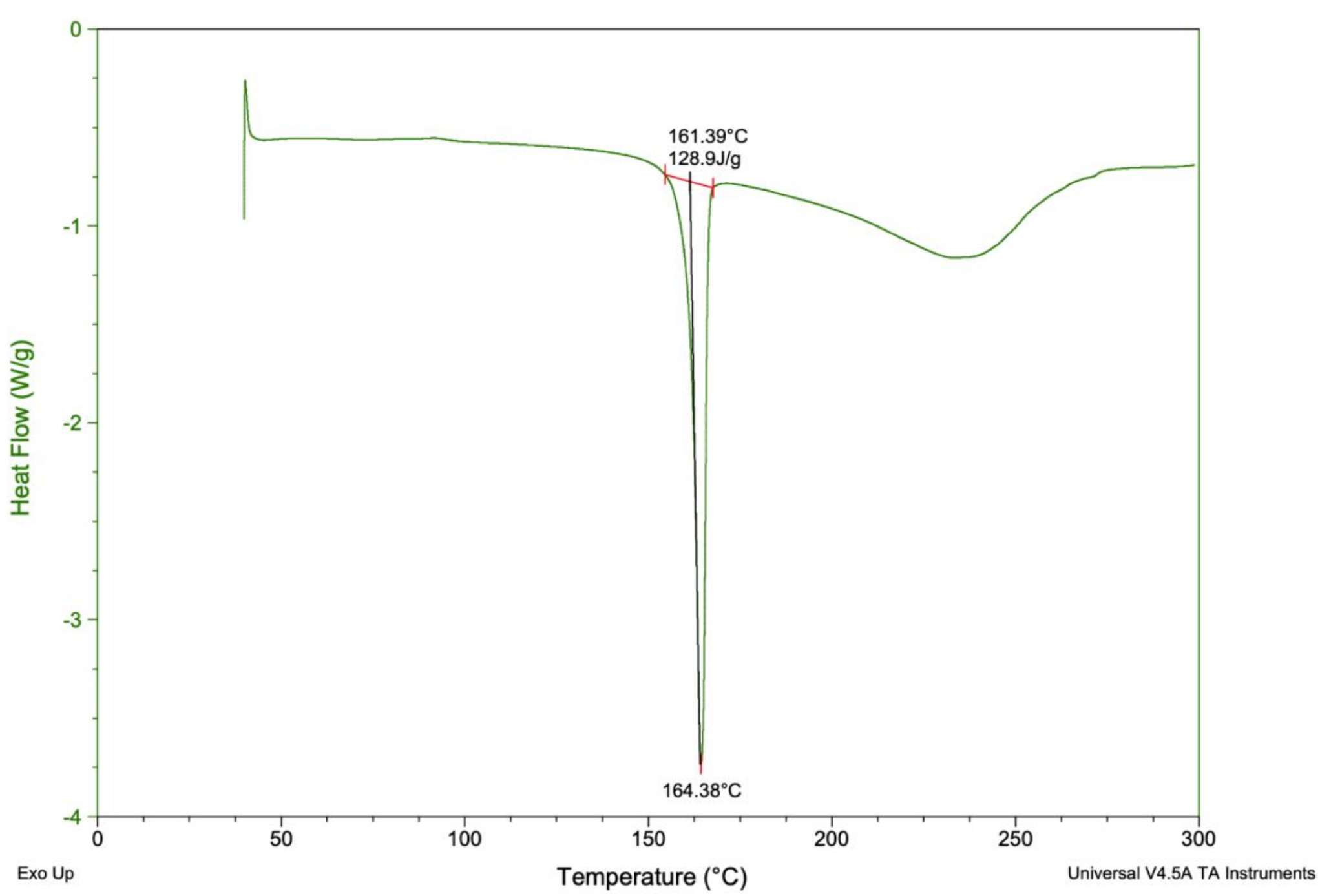

Fig S13: DSC data for (a) oP^4(OH)$_2$ and (b) oP^6(OH)$_2$

References

1. Kirinda, V.C., Schrage, B.R., Ziegler, C.J. and Hartley, C.S. (2020), ortho-Phenylene-Based Macrocyclic Hydrocarbons Assembled Using Olefin Metathesis. Eur. J. Org. Chem., 2020:5620-5625. https://doi.org/10.1002/ejoc.202000950

2. Craig, A. F.; Clark, E. E.; Sahu, I. D.; Zhang, R.; Frantz, N. D.; Al-Abdul-Wahid, M. S.; Dabney-Smith, C.; Konkolewicz, D.; Lorigan, G. A. Tuning the Size of Styrene-Maleic Acid Copolymer-Lipid Nanoparticles (SMALPs) Using RAFT Polymerization for Biophysical Studies. Biochim. Biophys. Acta (BBA)-Biomembranes 2016, 1858 (11), 2931–2939.
